# Designing a context-specific intervention in practice: learning from a school-based physical activity intervention

**DOI:** 10.3310/nihropenres.14336.1

**Published:** 2026-06-30

**Authors:** Danielle House, Abi Howard, Simona Kent-Saisch, Alice Porter, Tom Reid, Roger Morbey, Lydia Emm-Collison, Frank de Vocht, Ruth Salway, Russell Jago

**Affiliations:** 1 Population Health Sciences, University of Bristol Medical School, Bristol, England, UK; 2 NIHR Bristol Biomedical Research Centre, Bristol, England, UK; 3 Centre for Exercise, Nutrition & Health Sciences, University of Bristol, Bristol, England, UK

**Keywords:** physical activity, public health, interventions, schools, context, settings, complex interventions

## Abstract

**Background:**

Public health interventions need to be designed for the intervention setting. Traditionally, to maximise internal validity, school-based pupil physical activity interventions deliver an identical programme across all settings. However, an interventions’ success will depend on the context in which it is implemented, i.e. a schools’ social, cultural, economic, environmental and other characteristics. In this paper we use the PASSPORT (Physical Activity via a School-Specific PORTfolio) study as a case study for designing a context-specific public health intervention. We used a portfolio approach that allows schools to select and deliver tailored intervention content that is most suitable to their setting. We outline how we developed the intervention and draw out key learning from this for public health interventions generally.

**Methods:**

The PASSPORT intervention was developed across multiple research steps that were designed to progressively build key knowledge. Formative studies included: a scoping review of intervention components; school staff interviews; a rapid ethnography in three primary schools; and a school survey. These then informed the intervention design and content, which were developed via two mechanisms: the co-design of the intervention tool with primary school staff and by centring school expertise and experience in intervention design and content. Finally, we conducted a pilot study to learn from both the delivery of the intervention and associated evaluation data collection.

**Key learning:**

Key learning from the intervention design and development has been gathered around four areas: Ground intervention design in a range of data and insight, centre user experience, design for intervention flexibility and user autonomy, and, build in processes to test and iterate.

**Conclusions:**

Each of the formative studies and the intervention design steps provided parts of the complex picture of intervention context and design needs. Combined, the results of the studies supported the development of a context-specific intervention.

## Plain Language summary

**What this paper is about:**

Programmes designed to support people’s health need to be appropriate for the specific community they serve. For example, schools are a great place to support lots of children to be more active, but a programme needs to fit into the day-to-day life of a school for it to work well. Usually, health programmes are the same in each setting. But each setting is different, with different resources, people, equipment, and cultures. In this paper we describe how we designed a flexible programme that would be different in each school setting. We outline the steps we took to develop the programme and draw out key learning from this, to help other researchers who want to develop similar programmes.

**What we did:**

We did several smaller projects to build up the information we needed to develop the flexible programme. These are described in the paper and include looking at current academic literature, doing interviews with school staff, spending lots of time in three schools, and doing a survey with many schools. We then took our learning from these projects to design the programme in workshops with school staff, and we used school knowledge and staff to develop the activities that make up the overall programme. We then tried out the intervention in two schools to learn from this.

**What we learnt:**

We learnt several key lessons that would be useful for other researchers who want to design a similar programme. 1) It was useful to have such a range of information to bring into the programme. 2) It was important to have the users, the people who would deliver the programme, at the centre of the programme development. 3) Designing the programme to be flexible was helpful, so that it could be different in each setting, and it was good for schools to be the decision-makers in this. 4) It was necessary to try out and improve the programme lots of times.

## 1. Introduction

Public health interventions should be designed for their setting, addressing the needs of the people they are impacting and the systems they work within. ^1, 2^ It is increasingly recognised that this means intervention settings should be viewed as complex adaptive systems, and that interventions in turn must adapt to that complexity to be successful. ^3^ In children’s physical activity research, school-based interventions have great potential to support large numbers of children to be active. ^4– 6^ However, despite a small number of exceptions, research has shown, in general, that these interventions have not increased children’s moderate-to-vigorous intensity physical activity (MVPA). ^7– 13^ This is a critical issue, as physical activity is essential to children’s physical and mental health and wellbeing. ^14– 16^ However, the majority of children are not meeting the recommended minimum levels of physical activity ^17, 18^ in the UK or globally. ^19– 22^ Traditionally, school-based pupil physical activity interventions (as with many public health interventions) take a ‘one-size-fits-all’ approach, designed to deliver an identical programme across all schools or settings with a focus on internal validity. A key aspect that is assumed independent from the active component of an intervention in this approach is the context in which it is implemented, i.e., a schools’ social, cultural, economic, environmental and other characteristics. ^23^ We have suggested that this could be a key factor in the limited success of school-based physical activity interventions, ^23^ and can contribute to low external validity and a lack of intervention sustainability and success when scaled up. ^24^ Contextual variation in school-based interventions should be no different from real-world interventions implemented in other environments. Therefore, this is potentially an issue for public health interventions generally, beyond school-based physical activity interventions.

The PASSPORT (Physical Activity via a School-Specific PORTfolio) project aims to develop and evaluate a context-specific school-based physical activity intervention that attempts to address the problem outlined above. The central question at the outset of this study was to explore and establish what was meant by ‘context’ in relation to primary school pupil physical activity, and what a ‘context-specific’ intervention design could look like. ^23^ We hypothesised that an intervention where content is optimised to its local context would maximise impact on pupil physical activity outcomes and could have population level impact. One way we could design a context-specific intervention is with a portfolio approach that allowed schools to select and deliver tailored intervention content that would be most suitable to their setting.

In this paper we use the development of the PASSPORT intervention as a case study for designing context-specific interventions. To achieve this aim, this paper has four key elements:

1. To provide a brief description of the PASSPORT intervention
2. To outline how the context-specific intervention was developed
3. To outline how the context-specific intervention was pilot tested and the associated findings
4. To highlight how the learning from this project can be applied to context-specific intervention development in public health generally.

*Ethical approval*

All studies with participants outlined in this paper received ethical approval from the University of Bristol Research Ethics Committee. Their individual references are: school staff interviews (Ref: 15866 19/10/2023), rapid ethnography (Ref: 16095 18/01/2024), school survey (Ref: 20041 06/08/2024), co-design workshops (Ref: 20957 23/08/2024), and pilot study (Ref: 23839 11/03/2025).

*Consent*

Across these studies informed consent or assent was obtained from all participants. ^25^ All participating schools signed school study agreements. All school staff and wider school community members provided written consent to participate as an individual in a research activity, with the exception of observational data collected in the rapid ethnography, where lead staff members provided verbal consent. This was deemed most feasible in consultation with schools, where observation of lessons is a day-to-day practice. All school staff were provided with study information sheets, consented to arrange an observation, and consented again for the observation to go ahead. In our studies that involved minors, parents provided written consent and pupils written assent. Pupil participants in the creative research activities, part of the rapid ethnography, provided written assent on paper at the start of the activity. Pupil participants in the pilot study provided written assent via the family consent form, through which parents and children consented to participate in the study.

*Patient and public involvement*

School staff, parents, and pupils have been involved in each study described in this paper from the design stage, commenting on and shaping into data collection plans, information sheets, topic guides and other communication and recruitment processes and materials. As part of the context-specific approach we have taken to intervention design, school staff experience was brought into the research team to lead this work. The intervention was primarily developed through co-design workshops with school staff. Intervention content was further reviewed by primary school PE expertise. And extensive engagement with staff, parents, and pupils was undertaken in the pilot study to inform our recruitment processes. This public engagement is described and detailed throughout the paper, as it played a central role in the development of the context-specific intervention.

## 2. The PASSPORT intervention

The PASSPORT intervention is a flexible, school context-specific portfolio whereby primary schools select a bespoke physical activity programme from a range of recommended activities that builds on their current strengths, acknowledges limitations of the school setting, and identifies opportunities to introduce physical activity across the school day. A digital PASSPORT ‘tool’ supports staff to review and select their bespoke programme, as well as support their delivery of it. The intervention takes a whole-of-school approach to physical activity ^26^ and the intervention and implementation support strategies are aligned to the Consolidated Framework for Implementation Research (CFIR), ^27, 28^ to ensure it addresses key determinants of implementation in primary school settings.

The PASSPORT intervention (outlined in Figure 1) has three main steps: 1) schools complete a baseline context assessment to provide information on their physical activity environment and practices. The context assessment captures the current provision across the school day, as well as their facilities, policies, organisational structure, and physical activity culture. 2) This information is used by the research team to recommend activity options from the portfolio that work for that school, via the tool. 3) Schools use the tool to select and deliver their bespoke programme from the recommendations. To support the delivery of bespoke programmes, a core package of resources support the development of school physical activity culture, staff pedagogy and skills, and alignment to school systems and priorities. The core package resources are identical in all schools.

**Figure 1.**
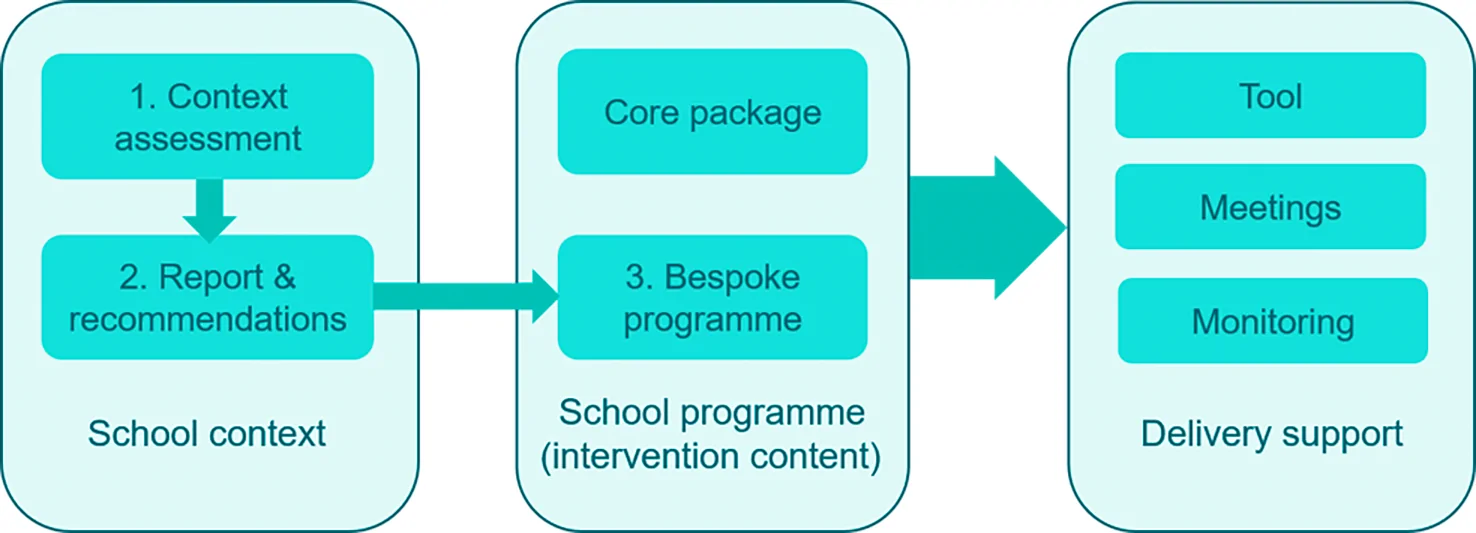
Flow of the PASSPORT portfolio intervention.

The content for the portfolio (i.e. the possible activities schools are recommended and build their bespoke programmes from) is designed along six pathways that cover physical activity opportunities across the school day and whole school community ( Figure 2). These pathways are: *Physically Active and Outdoor Learning; Active Breaks; Breaktime and Lunchtime; Active Travel; Before and After School Activities;* and *School and the Wider Community.* Schools are recommended activities from priority pathways identified through their context assessment, and activities are designed to build, establishing foundational steps that can be expanded on and deepened. Schools deliver their programmes with the support of PASSPORT staff and the ongoing use of the PASSPORT tool, which has budget, action plan, and milestone tracker functions.

**Figure 2.**
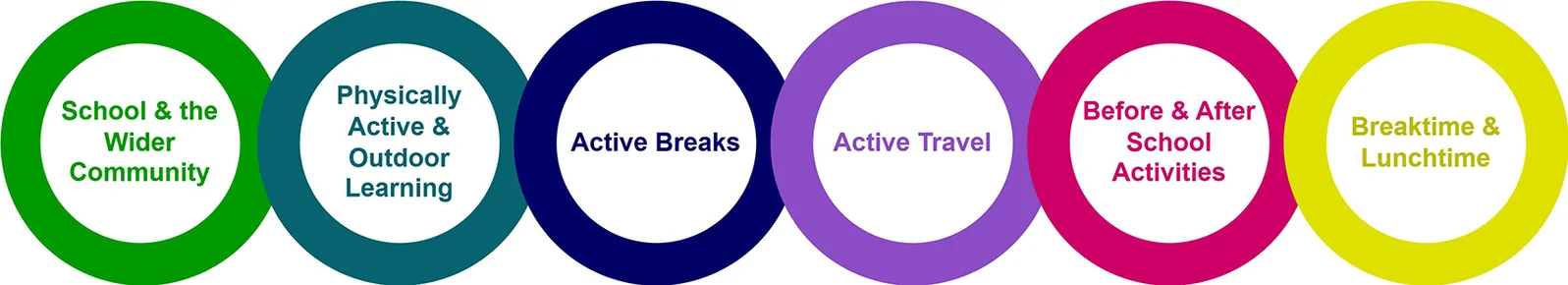
The PASSPORT physical activity pathways (portfolio content).

## 3. Developing the PASSPORT intervention

The PASSPORT intervention was developed and iterated across multiple work packages, each designed to establish necessary knowledge that progressively built. This section describes each of these work packages and how it contributed to the development of the context-specific intervention. *3.1) Formative work* describes: a scoping review of intervention components to inform the portfolio content; school staff interviews to understand school context for physical activity as well as feasibility and acceptability of the intervention; a rapid ethnography in three primary schools to deepen our understanding of school systems; and a school survey to understand school physical activity at a broader scale and explore the use of a survey method to capture this. *3.2) Intervention design and content development* describes: the co-design of the intervention tool with primary school staff, and; how we centred school expertise and experience in intervention design and content. Figure 3 provides an overview of the development of the PASSPORT intervention.

**Figure 3.**
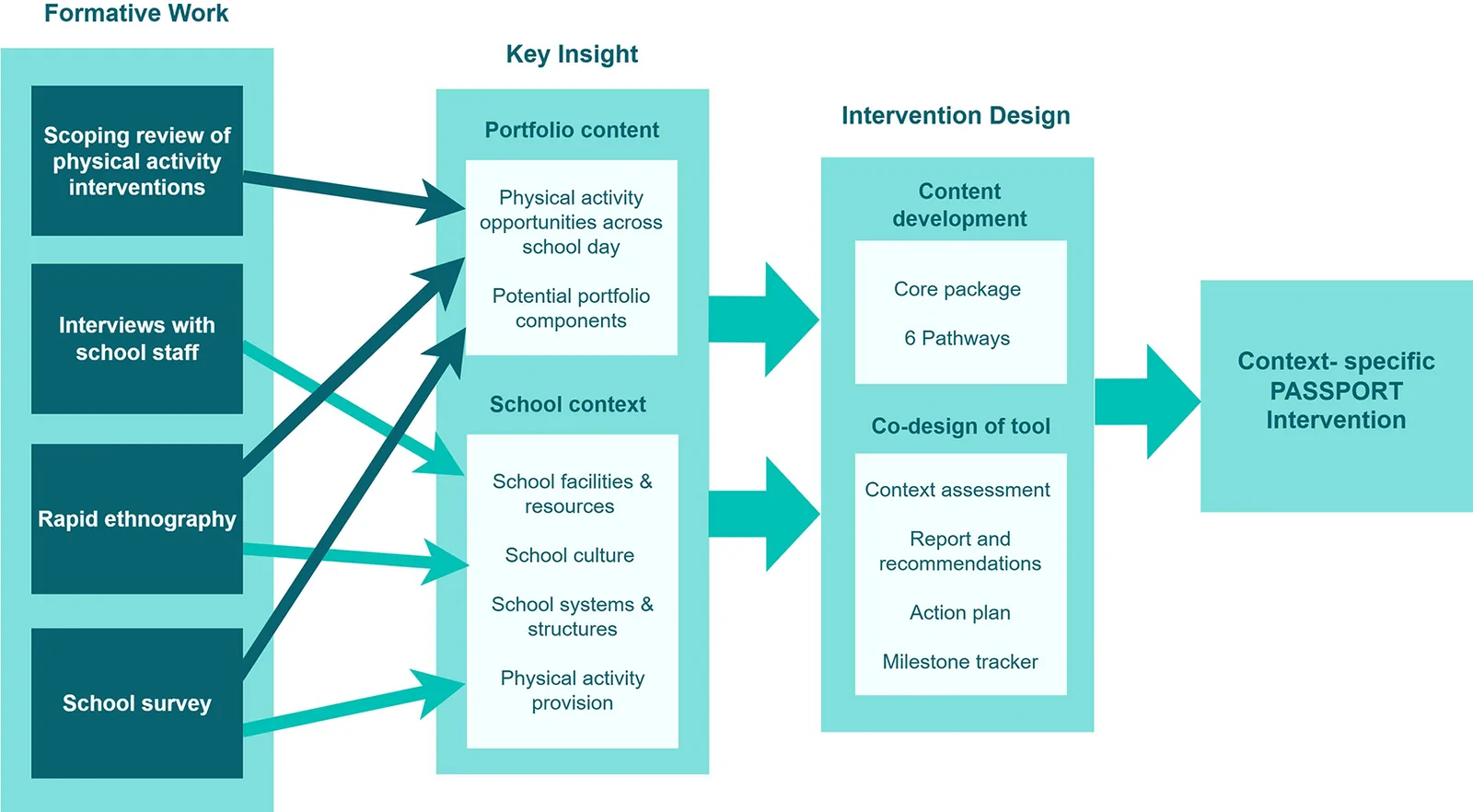
Development of the PASSPORT intervention.

### 3.1 Formative work

The purpose of the formative work for the PASSPORT intervention was to establish the meaning of ‘school context’ in relation to pupil physical activity, and to have the necessary data and insight to develop an intervention that would be useful and suitable for schools. Key to the formative work was to use a variety of methods capturing a wide range of perspectives. Combined, this work provided a complex but consistent picture of the aspects of primary school context that are key for pupil physical activity, and a strong sense of the policy and structural/organisational context of schools for whom we were designing the intervention.

*Scoping review of physical activity interventions in primary schools*

We conducted a scoping review to identify previous school-based physical activity interventions that could form the basis of the PASSPORT intervention content. ^29, 30^ As we were focussed on the whether it was feasible to deliver each intervention component it was not a requirement that an intervention had been shown to have positively impacted on physical activity. This is important because it may be that an intervention did not achieve a statistical impact on physical activity in all schools because the context was different in each school. The program could still be useful in the right context. European school-based intervention studies aimed at increasing physical activity in children aged 7–11 years published in English since 2015 were included in the review. 79 articles were included, constituting 45 intervention studies. We identified 177 intervention components, which were synthesized into a framework of 60 intervention component types across 11 activity opportunities: six within the school day, three within the extended school day and two within the wider school environment. These intervention components, and particularly the opportunities for activity across the school day, guided the other formative studies and formed a basis for the development of the PASSPORT intervention content, as a first iteration of the pathways.

*Semi-structured interviews with school staff*

We conducted a study of semi-structured interviews with school staff to establish a foundational understanding of ‘school context’ for pupil physical activity. These interviews explored the elements of school context that impact pupil physical activity and the feasibility and acceptability of a portfolio intervention design. We conducted 33 interviews with staff from 19 primary schools in the wider Bristol area ^31– 33^ between November 2023 and January 2024. Staff roles included headteacher/principal or deputy, class teachers, teaching assistants, Physical Education (PE) subject leads, dedicated PE teachers, and a Parent Teacher Association (PTA) chair. Schools were purposively-sampled with a range of sizes, locations, and sociodemographic characteristics. Full school and participant information and study processes are reported in published papers. ^32, 33^ Analysis of these interviews brought out the role of facilities and resources for pupil physical activity in primary schools, ^32^ and the role of social context, i.e. the organisational, political, cultural, and sociodemographic characteristics of a school. ^33^

*Rapid multi-site team ethnography*

In order to provide greater depth and perspectives from within a school system, we conducted a rapid, multi-site team ethnography in three purposively sampled Bristol primary schools between March and July 2024. ^34– 36^ This approach prioritises understanding structures, decision-making processes, and culture, as well as the experiences of people, within the settings. ^37^ Documentary, conversational, and observational data collection captured multiple perspectives from the school communities over time, which enabled comparison across the schools, roles, and pupils. The focus for data collection activities was informed by the interview study and scoping review. Participants included staff that have a role in delivering physical activity or broader health programmes, or governance and decision-making, as well as broader school community members such as external providers of active clubs and PE, school Governors, or PTA members. Data on physical activity provision, spend, and participation as well as school demographics were collected and informed further data collection in each school. Observations were conducted throughout the school day to understand when, where, and how pupils were active and where any potential opportunities for physical activity and the intervention lay. Additionally, school meetings and events were observed to understand broader school structures and priorities. Interviews were conducted with a range of key community members in each school, and creative research activities were undertaken with Year 5 pupils to bring in their perspectives and experiences. Detailed information on all study schools, participants, and processes are reported in published papers. ^35, 36, 38^ This rapid ethnography deepened our understanding of school physical activity and school systems, which provided several implications for our intervention design. ^35^ Across these three schools there were areas of convergence and divergence on pupil physical activity. This highlighted consistent features we could build on in the intervention (e.g. consistency across the roles of different school communities for physical activity), but also where flexibility and difference were key to an acceptable intervention (e.g. building on the schools’ idea of success for physical activity in their school, or the need to provide multiple opportunities across the whole school day to meet the needs of every school context).

*School survey*

Finally, we conducted a survey to collect data on school physical activity provision, policies, and readiness for change among a broader sample of schools. With this we also aimed to understand how much context differed between schools, and to trial questions to be included in the school context assessment. The school survey was conducted across the wider Bristol area with responses collected between September and December 2024. ^39^ Primary school staff who were either PE leads, dedicated PE teachers, or members of the school Senior Leadership Team (SLT) were asked to complete the survey. The questionnaire consisted of four sections: 1) How physical activity is provided and promoted to pupils outside of PE lessons; 2) The perceived quality of pupil physical activity provision and promotion outside of PE lessons; 3) School culture towards physical activity and level of priority physical activity is given within schools; and 4) School-level readiness to change regarding pupil physical activity provision and promotion. We received 59 unique school responses from 311 schools who were invited to participate (19% response rate). Table 1 presents the demographic characteristics of the schools and participating school staff in the sample. The majority of participating schools were located in urban areas (76%), from areas of lower deprivation (59% with Index of Multiple Deprivation ^40^ of 7–10) and with below national percentages of pupils eligible for free school meals (78%). ^41^ Most school staff completing the survey were teachers with a PE lead role.

**Table 1.** School survey participating school and staff demographic characteristics (N = 59).

School characteristics	n	%
*Urban/rural classification*		
Urban	45	76%
Rural	14	24%
*School Region*		
City of Bristol	23	39%
South Gloucestershire	16	27%
North Somerset	8	14%
Bath and North East Somerset	12	20%
*Free School meal* ^1^ *%*		
Below national percentage eligible	46	78%
Equal to or above national percentage eligible	13	22%
*School postcode Index of Multiple Deprivation* ^2^		
1–3 (most deprived)	12	20%
4–6	12	20%
7–10 (least deprived)	35	59%
*Number of pupils in school*		
0–200	15	25%
201–400	24	41%
400+	20	34%
*School Management*		
Local Authority Maintained	22	37%
Academy	37	63%
**Participant characteristics**	**n**	**%**
*Participant roles*		
Senior leadership team member only	12	20%
Senior leadership team member and additional role(s) (i.e., PE lead)	5	9%
Dedicated PE teacher, PE Coordinator, or PE Lead	8	14%
Teacher and additional role(s) (i.e., PE lead)	27	46%
Teacher only	5	9%
Other	2	3%

^1^ a UK government scheme to provide free school meals to children from low income families, data from academic year 2023/24 when 24.6% of pupils nationally were eligible for free school meals ^41^

^2^ a measure of area deprivation, with 10 being least deprived and 1 being most deprived ^40^

The school survey highlighted variation across schools in physical activity provision, culture, and readiness for change, suggesting that a contextually-sensitive approach to a physical activity intervention was necessary, i.e., each school had different needs and areas for development. For example, overall, school staff reported their perceived quality of their physical activity provision and promotion was good, however there was varying agreement in areas such as whether their physical activity clubs were oversubscribed and whether it was difficult to timetable pupil physical activity opportunities across the school day ( Table 2). Schools reported a variety of areas in which they planned to spend their PE and Sports Premium funding, with external providers (88% of schools), new equipment (78% of schools), resources/materials (71% of schools), and teaching training (61% of schools) most frequently selected. However, all possible areas of spending had a strong response rate, with subsidising club fees lowest (42% of schools), suggesting all areas needed to be included as options in the PASSPORT intervention. These findings and others informed areas of focus for the intervention content, but of key importance the school survey, combined with previously used measures of school physical activity environments, ^42, 43^ formed a basis of the school context assessment tool. School survey questions and results were reviewed to identify key items to include in the first draft of the context assessment. Survey items were included based on their relevance and importance to understanding school physical activity context and how schools had responded. For example, questions were not included if the answers were very similar across all schools and thus did not help distinguish between different contexts.

**Table 2.** School survey perceived quality of pupil physical activity provision and promotion (N = 59).

	Strongly agree	Agree	Neutral	Disagree	Strongly disagree	Don’t know	N/A
The opportunities for physical activity are inclusive to all children in our school	42%	49%	7%	2%	0%	0%	0%
The facilities and equipment used for physical activity are inclusive to all children in our school	31%	59%	7%	3%	0%	0%	0%
Our sports and play equipment (e.g. rackets, balls, bibs, games) are high quality	12%	61%	24%	3%	0%	0%	0%
The national or local programmes/initiatives that our school takes part in have been worth doing to increase the physical activity levels of our pupils	17%	53%	24%	0%	2%	3%	2%
The training and resources school staff have been provided with makes them feel confident to support pupil physical activity	10%	56%	19%	8%	3%	2%	2%
Our physical activity facilities (e.g. playground, fields, courts, indoor spaces) are high quality	15%	41%	15%	19%	8%	2%	0%
Physical activity clubs at our school engage less active/less sporty pupils	5%	39%	22%	32%	0%	2%	0%
In our school, timetabling pupil physical activity is difficult to do within the school day	15%	29%	3%	36%	15%	2%	0%
In our school, timetabling pupil physical activity is difficult to do before or after school	5%	24%	8%	42%	17%	2%	2%
The physical activity clubs (e.g. before school, lunchtime, after school) at our school are over-subscribed.	8%	20%	36%	32%	2%	0%	2%

### 3.2. Intervention design and content development

The next step was to use data and insight from the formative work to undertake a structured approach to intervention design and content development. Key to this was centring school expertise and experience in these processes, to best support the development of an intervention that understood and was designed for school context.

*Co-design of the PASSPORT tool*

The PASSPORT tool that schools use to assess their context, review and select a bespoke programme, and support programme delivery, was developed through a co-design process with primary school staff, ^44^ to ensure that it was shaped by the experience and insight of the potential users. We held three ideation workshops ^45^ between November 2024 and May 2025. Workshop 1 had 10 attendees, Workshop 2 had 10, and Workshop 3 had 6. This was a total of 11 staff from 11 local primary schools, including head teachers (n = 2), dedicated PE teachers (n = 3), class teachers with a PE lead role (n = 5), and class teachers (n = 1). We used design principles to structure and guide these workshops, ^46, 47^ following the double diamond design process ^48^ and applying user-centred design methods described below. The workshops explored and validated the data and insight from formative studies, tested and refined the context assessment, and iterated prototypes of the tool. Figure 4 outlines this design process.

**Figure 4.**
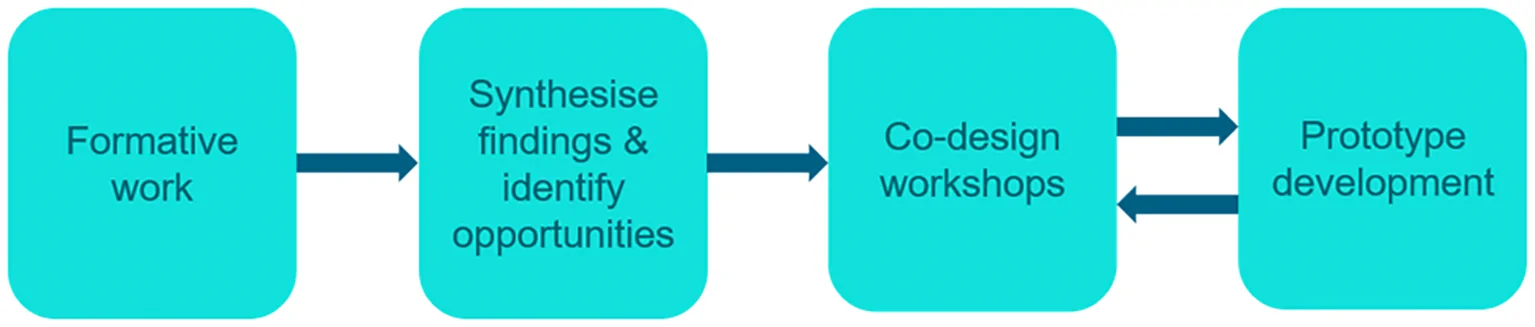
Overview of the co-design process.

In Workshop 1 (in-person), group members validated and scrutinised our understanding of the school system, different staff priorities, and the general role and landscape of physical activity in primary schools, through several activities. We used insight from the formative work to develop user personas for key school staff and pupils, which workshop members read, annotated, challenged, and validated ( Figure 5), ensuring we understood the position and priorities of each user group. We also used the formative work to create opportunity statements that highlighted key opportunities for supporting pupil physical activity in school which workshop members were asked to prioritise by most important. Improving participation of all pupils and embedding a physical activity culture were identified as areas of highest priority. Members then developed initial design ideas and features for the tool via paper prototyping activities, exploring how information could be organised and the level of detail necessary for decision-making when selecting activities, such as cost, time resource, and target audience ( Figure 6). Following Workshop 1 we developed prototypes of each part of the tool, and the idea of the core package of resources to build physical activity culture and staff also emerged at this stage.

**Figure 5.**
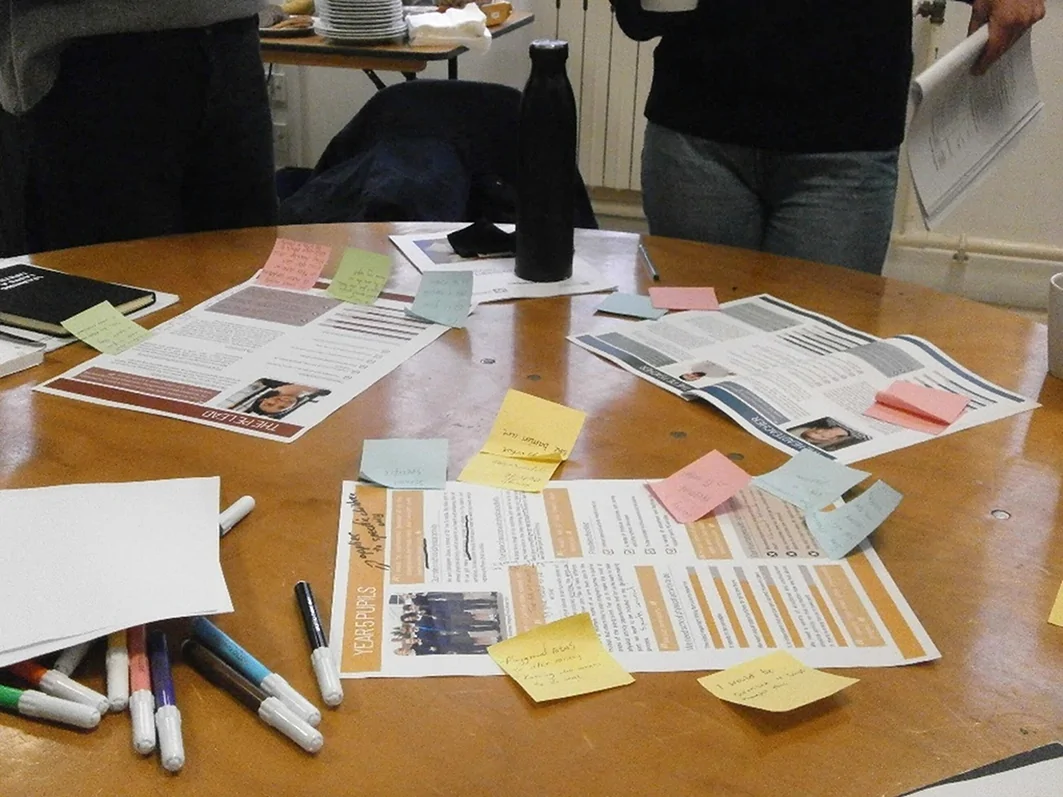
Workshop 1 persona group work.

**Figure 6.**
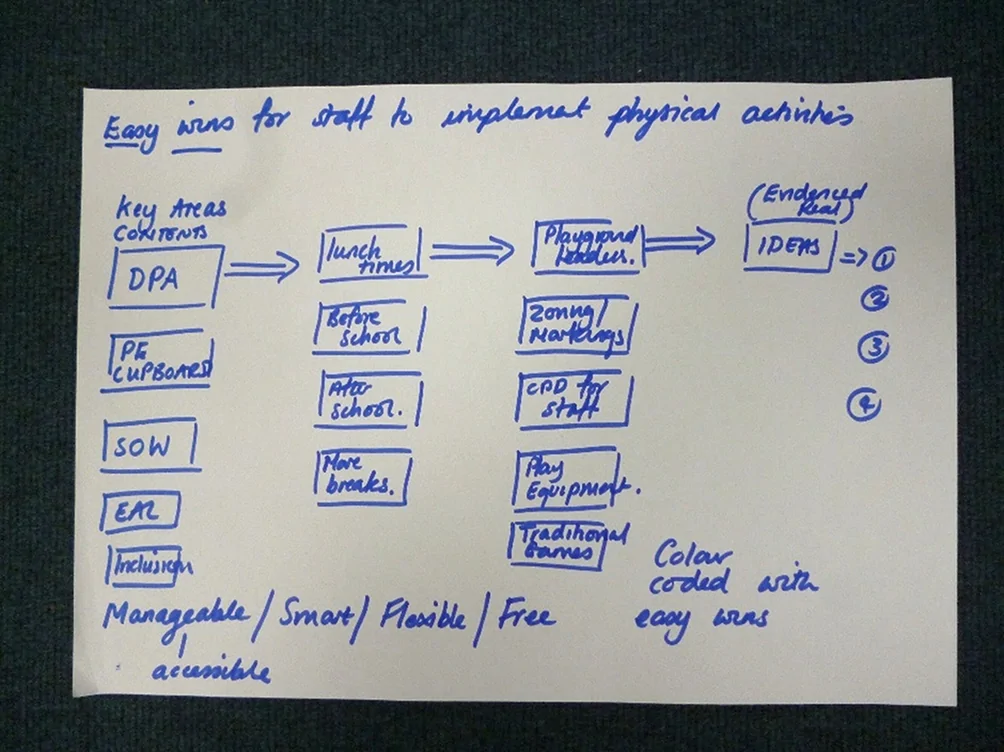
Workshop 1 initial ideas for the tool.

Workshop 2 (in-person) further explored issues and ideas that were identified in Workshop 1 and the core package was introduced, as well as logic models for using the tool. School physical activity culture was explored through a prioritisation activity, and members engaged with several general questions about intervention design and content that had arisen between the two workshops. Members then completed and refined a draft context assessment on paper, advising on what needed to be kept, removed, and expanded on in order to maximise the usefulness for the tool and for schools broadly. Finally, the group explored the updated paper prototypes which they iterated and refined ( Figure 7). The importance of aligning the tool and content to key policy frameworks was communicated by workshop members, namely the Ofsted inspection framework, the national curriculum for PE, and the PE and School Sport Premium, and that this would support schools in a practical and meaningful way to meet their existing demands and reporting requirements. Design features, such as how and where to best include different staff in using the tool, and the inclusion of an action plan, also arose from this workshop. Following Workshop 2 we refined the paper prototypes of the tool and draft context assessment. The context assessment was then translated to Online Surveys ( https://onlinesurveys.jisc.ac.uk/) and a digital wire-frame prototype of the tool was developed in the design software Penpot. ^49^

**Figure 7.**
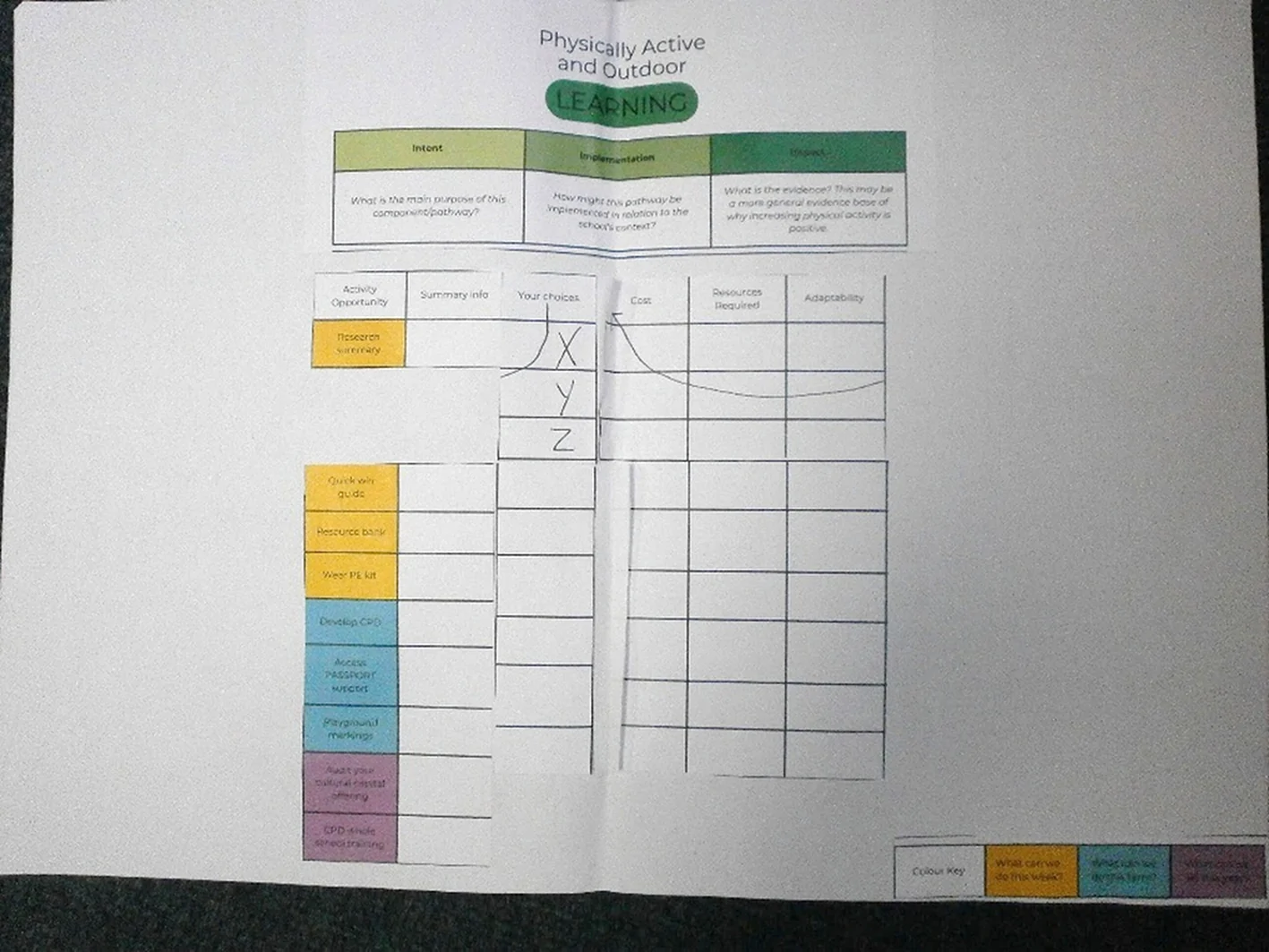
Workshop 2 paper prototyping group activity.

In Workshop 3 (online), members were guided to interact with and ‘use’ the digital prototype, including completing the context assessment digitally and giving feedback on the tools’ design, user journey, and content ( Figure 8). After Workshop 3 the prototype tool was further refined and iterated based on feedback, and the inclusion of a milestone tracker function was added. Then, for use within the scope and limits of the PASSPORT study, we translated the Penpot prototype into a useable system within the MS Office package.

**Figure 8.**
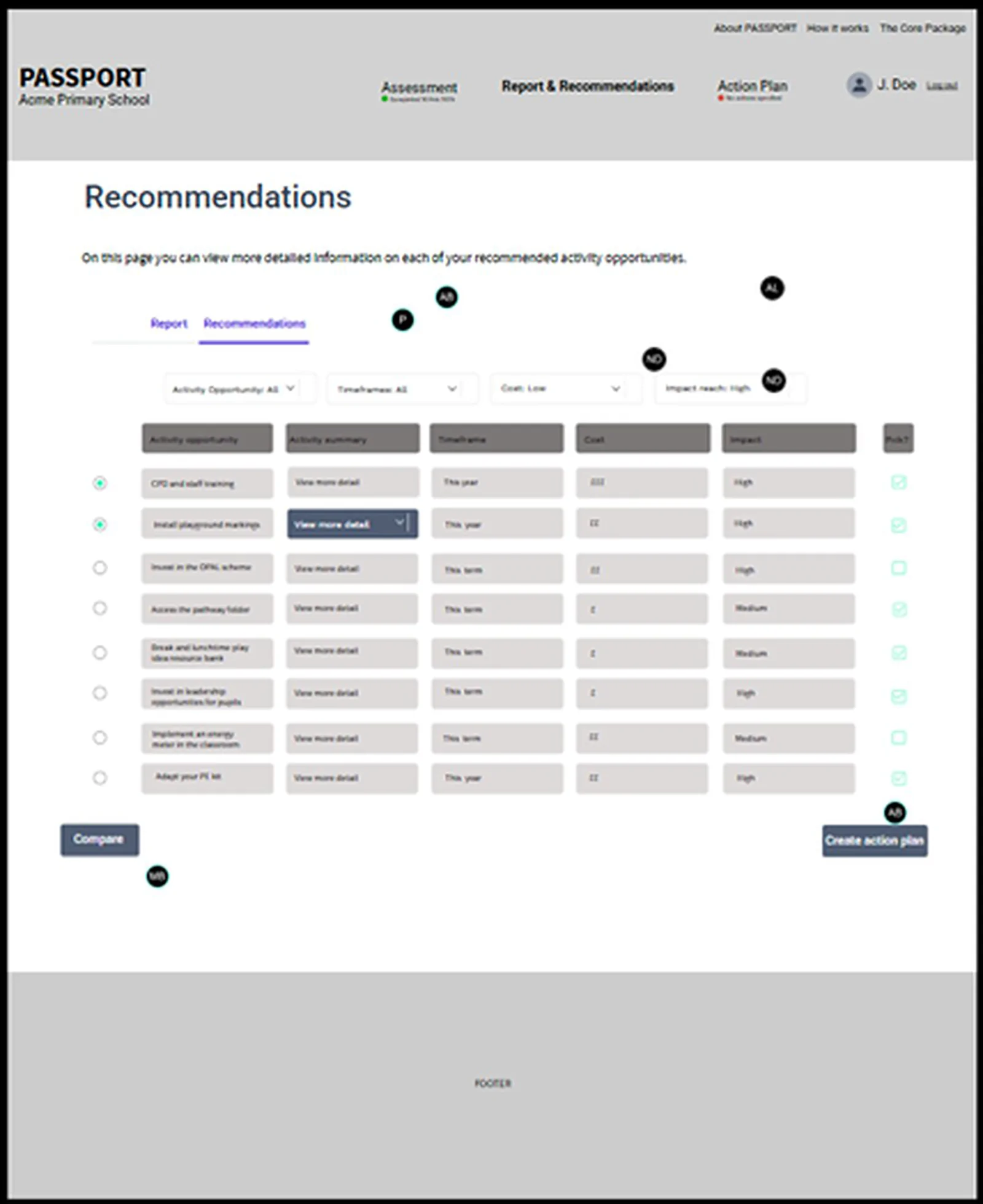
Workshop 3 screenshot from the digital prototype.

Additionally, workshop activities and conversations informed the portfolio content, i.e. the activities that would be recommended to schools through the tool. Members helped to refine content to the six pathways (see Figure 4) and emphasised how all content needed to provide options for pupils with Special Educational Needs and Disabilities (SEND) and be adaptable to all contexts.

*Physical Education and school expertise in content development*

Alongside the development of the tool, content was required for the portfolio, i.e. each of the activities that could be recommended across all six pathways. To maximise the relevance of this content to schools, an experienced PE teacher joined the study to lead this work and provide school PE and physical activity expertise, system understanding, and language, as well as lead intervention delivery support. Informed by the formative studies and co-design process, desk research was undertaken to map out activities across the six pathways and develop the materials and resources required for a school to implement each activity. For additional input and scrutiny, all draft content was reviewed by a primary PE specialist and feedback integrated. The Consolidated Framework for Implementation Research (CFIR) was used to further strengthen the intervention content. ^27, 28^ CFIR provides a structure to consider factors that influence implementation, including characteristics of the intervention, the school environment, external influences, individual staff factors and the processes used to support change. Using this framework helped ensure that implementation was embedded within design decisions rather than treated as a separate phase. Lastly, a School Advisory Group of teachers, head teachers, and PE leads advised on aspects of our planning and design and provided comment on any documents, queries, or contextualising that was necessary.

## 4. Piloting the PASSPORT intervention

To pilot and refine the PASSPORT intervention, a study was conducted with two contextually contrasting schools (inner city and suburban, differing pupil demographics) in the Bristol area as learning partners over a seven-month period (June 2025 – December 2025). ^50^ This pilot was also used to pilot and improve plans for school and pupil recruitment, communications, and physical activity data collection for a main evaluation, and so did not aim to estimate the impact of the intervention on physical activity outcomes.

*Pupil recruitment and quantitative data collection*

Alongside the intervention delivery and evaluation, physical activity data were collected from pupils to monitor recruitment and retention rates, and amount of valid data. Parent consent and pupil assent was provided, along with pupil demographic information. All Year 4 pupils were eligible to participate, and physical activity data were collected via wrist-worn Axivity AX3 triaxial accelerometers worn for six days on three occasions (once at T0, T1, and T2). Pupils completed a paper questionnaire in school time which they completed twice (T0 and T2). Pupil absence data was collected for the study cohort year group (T0 and T2). Data collection in the schools was successful, and demonstrated our plans a future trial and evaluation are feasible. A total of 46 pupils were recruited at baseline from a potential total of 57 across the two schools, with high levels of data provision across the three measurement points (64–85%). At each measurement point (T0, T1, T2), two weekdays of valid accelerometer data were required to calculate a pupil’s mean weekday MVPA. Nearly all (45; 98%) pupils provided a valid weekday MVPA from at least one measurement point and over half (25; 54%) had measurements for all three. Table 3 provides school and participant demographic information, and Table 4 summarises data provision by time point.

**Table 3.** Pilot study school and participant demographic information at baseline.

	School 1		School 2	
	n	%	n	%
*School data*				
% pupils in receipt of Pupil Premium	-	49%	-	40%
% girls	-	48%	-	63%
% non-white ethnicity	-	27%	-	95%
*Participant data*				
Total pupils in year group	27	100%	30	100%
Total pupils recruited	17	63%	29	97%
Pupil Premium *	4	33%	8	30%
Pupil Premium missing/prefer not to say	5	29%	3	10%
Girls *	9	64%	10	53%
Gender missing/prefer not to say	3	18%	11	37%

* excluding missing data

**Table 4.** Pilot study data provision.

	T0 Baseline (Term 6 24/25)		T1 (Term 1 25/26)		T2 (Term 2 25/26)	
	n	%	n	%	n	%
Pupils recruited	46		45		46	
Pupil questionnaires returned	45	98%	-		46	100%
Valid wellbeing scores	37	80%	-		39	85%
Accelerometer measurements	41	89%	40	89%	34	74%
Weekday MVPA * scores (which included at least 2 weekdays)	40	87%	35	78%	30	65%

*Note*: in School 2 one pupil left after baseline (T0) and an additional pupil was recruited T2.

* MVPA = moderate-to-vigorous physical activity

*Intervention and evaluation*

Schools completed a school-study agreement to consent to the study, which included providing school demographic data. At T0 (baseline, Term 6 of the 2024–25 school year, June–July 2025), both schools completed the context assessment and received their reports and recommended activities via the PASSPORT tool, as well as the core package resources. At T1 (Term 1 of the 2025–26 school year, September–October 2025) each school received £3,000 to spend on their bespoke programme, as well as their action plans and milestone trackers via the tool. They began delivery of their bespoke programmes which continued into T2 (Term 2, November–December 2025). A process evaluation was conducted to understand implementation and user experiences and further improve the overall intervention. Semi-structured interviews with intervention leads (in both schools this was the Head Teacher) at T0 and T2 (four interviews in total) explored experiences of using the tool and selecting their programme, delivering the programme, any successes or barriers, and key learning for us to take forward to improve the intervention. At the start of T2, schools completed an intervention implementation form to provide an update on progress, thoughts about the programme and the support given, as well as the response of the wider school staff and pupils. At the end of T2 schools completed an intervention component form to assess their bespoke programme, associated costs, and the engagement of staff and pupils.

*Findings*

The delivery of the intervention and use of the PASSPORT tool was well received. Quotes have been used to illustrate points in the text below. These quotes are identified by the school ID (S1, S2), data collection method (interview = I, intervention implementation form = IIF, and intervention component form = ICF), and where interviews, the interview number (I1, I2). For example, S1I2 is school 1 interview 2, or S2IIF is school 2 intervention implementation form.

Both schools made progress on the delivery of their bespoke programmes, although progress varied between the schools. The intervention implementation forms indicated that after one term of implementing their bespoke programmes (the start of T2), schools were still in the early stages, but delivery was progressing, with early engagement from pupils and staff alongside organisational and contextual factors that shaped the pace of implementation. For example, one school noted that *“some staff have already begun to try […] movement breaks in classrooms,”* (S1IIF), and they were in the planning stage for further activities. Several factors were highlighted that influenced early implementation and explained some difference between the schools, including competing priorities for school staff and practical challenges linked to environmental changes such as school procurement rules.

Interviews indicated that the intervention process, including completing the context assessment and using the tool to select and deliver their bespoke programme, was generally viewed as acceptable and appropriate. The context assessment was perceived as manageable and useful for reflection, with one participant noting that it *“didn’t […] feel really onerous”* and could help schools *“reflect on where you are or what your priorities are”* (S1I2). The programme selection part of the tool was also viewed positively, with one respondent describing it as *“easy to use … and not overly time consuming”*, adding that *“it flows absolutely fine”* compared with other reporting systems used in schools (S2I2). Participants spoke positively about the support provided by the research team, describing the overall process as *“a very supportive process throughout”* (S1I2).

Despite this, some implementation planning and support features of the tool were used less frequently than intended. Schools reported limited engagement with the action plan and milestone tracking elements because of competing demands on staff time. As one headteacher explained, *“you had to spend a bit of time doing it … it [lack of engagement] was more of a time thing”* (S1I2). Additionally, the intervention component forms showed mixed progress with allocating the programme budget which indicated that this could be better supported.

Participants described the activities recommended to them as practical and relevant to their school contexts, with one noting that *“they were all things that could be useful”* (S1ICF) and that the recommendations reflected areas where schools were already seeking to increase activity opportunities. The structured presentation of options was also viewed as helpful for decision making, with one headteacher explaining that the process helped move ideas *“from just having it in your head”* (S2I2) towards planned implementation. Pupils in school 1 were reported to respond positively to the opportunities for movement, and staff attitudes were generally supportive. The school reported one lunchtime activity was *“very inclusive … [and] suit children with a variety of needs”* (S1IIF). They explained that they saw early positive behavioural impacts from this activity, stating *“lunchtime incidents have dropped dramatically and there is less [pupil] dysregulation after lunch”* (S1IIF), and described it as engaged in by *“many children who weren’t otherwise as active”* (S1IIF).

Taken together, the responses suggest that the bespoke programme activities selected by the schools were suitable and appropriate to each school, and were integrated into existing school priorities and routines, with perceived benefits for pupil engagement and behaviour alongside increased opportunities for movement during the school day. The pilot and evaluation also brought out key learning and feedback to further develop the intervention. This included improving budget planning support in the tool at the activity selection and delivery stages, planning for an additional support meeting between the research team’s intervention delivery lead and a school to support using the action plan and milestone tracker, developing a video and information sheet to support the use of the tool, and the inclusion of questions to explore suggested areas for potential impact beyond MVPA in a future trial process evaluation, namely for pupil behaviour and attainment as well as attendance.

## 5. Key learning for designing context-specific public health interventions

Public health research is increasingly seeking to design complex interventions that are sensitive to different setting and community needs. This paper has outlined how the PASSPORT intervention, a school-based physical activity intervention, was developed, as a case study of designing a context-specific intervention. This section outlines key learning from the PASSPORT study to support putting context at the centre of public health intervention design broadly, beyond schools and physical activity. Table 5 summarises the key learning.

**Table 5.** Key learning from the PASSPORT study for designing context-specific public health interventions.

Key learning	Summary
Ground intervention design in a range of data and insight	Using depth and breadth in formative work that feeds into intervention development supports it to be context-specific by providing multiple steps of exploration and validation of ideas.
Centre user experience	Centring user experience in the design of a context-specific intervention works to ensure it will meet user needs, be suitable and appropriate, and be more likely to work within systems and priorities.
Design for intervention flexibility and user autonomy	Identifying where flexibility needs to be built in to the intervention design, and the balance of autonomy and guidance that is necessary at each step, centres context in the intervention by being responsive and adaptive.
Build in processes to test and iterate	The design of context-specific interventions requires not just evidence-based or co-design elements, but an interactive testing process to ensure that it works in practice and meets the needs of all involved.

*Ground intervention design in a range of data and insight*

Grounding the intervention development in a range of data and insight generated through the formative work and co-design supported context-specific design. For example, from the outset a central research question was what was meant by ‘school context for pupil physical activity’. Initial exploration of this in the interviews, deeper exploration in the ethnography, broader insights in the school survey, and then validation of the insights in co-design, established which aspects of schools needed to be considered and included in the intervention, which communities needed to be involved, and highlighted practical design and implementation issues such as how decision-making takes place and where physical activity sits within broader school priorities. As demonstrated in Figure 3, the interconnectedness of the data and insight has been key to understanding school physical activity context. None of the formative work studies or intervention development processes alone provide the whole picture, and the strength is in their combination. For example, the breadth and depth of the formative work enabled identification of which aspects of pupil physical activity diverge across school settings and which are broadly consistent, to feed into the intervention design. ^35^ Designing a context-specific, and in our case flexible, intervention is complex; creating a structured approach to capture this complexity and richness is valuable.

*Centre user experience*

Centring user experience in the design of a context-specific intervention works to ensure it will meet user needs, be suitable and appropriate, and be more likely to work within systems and priorities. Specifically, using user-centred design methods and processes carefully, and designing with school Physical Education and physical activity expertise (both within and outside of the research team), has made the PASSPORT tool more user friendly through understanding the level of information needed to use the tool effectively, how and where content needed to be adaptable, and which broader regulatory and statutory reporting systems it could support and align with. The co-design process of the PASSPORT tool brought forward insight and design decisions that would not have been apparent otherwise. Examples of this include: understanding the context assessment could meet a current gap in primary schools as a physical activity audit tool and expanding areas school staff identified; identifying the need for the core package to support school physical activity culture and the delivery of bespoke packages; designing for activities and areas of work to start with foundations and build, allowing a school at any stage in their physical activity provision to have relevant content; the importance of adaptability for pupils with SEND; and the addition of delivery tools such as the action plan and milestone tracker. Bringing context-specific expertise within a research team developing an intervention can provide further insight and contextual implementation support. Therefore, including user-centred design methods such as ideation workshops with key stakeholders, and bringing in contextual expertise, is valuable in developing public health interventions.

*Design for intervention flexibility and user autonomy*

A key mechanism for making the PASSPORT intervention ‘context-specific’ was to design a process for schools to select their own bespoke intervention, i.e. schools could have choice over a bespoke intervention that would differ in each setting. Across the development of the PASSPORT intervention school communities expressed their support for this premise, having experienced resources and programmes that were not be suitable for their particular setting. ^32^ A central question throughout the intervention design was identifying where flexibility needed to be built in and the balance of autonomy and guidance that was necessary at each step. For example, in the PASSPORT intervention completed context assessments are reviewed by a school physical activity expert in the research team and recommendations provided based on it. This is a structured process to focus priorities and avoid school overwhelm, but it takes place in conjunction with a meeting to discuss the recommendations, and schools can advise if adaptations are needed. Schools are then expected to select the activities they think are most suitable and ideal for their school, and therefore have autonomy over their choice, with support from the research team. Another area where flexibility has been intentionally designed is that the structure of the tool is easily extendable to accommodate additional intervention activities or pathways as the work develops. Designing for flexibility and user autonomy, therefore, centres context in the intervention by being responsive and adaptive.

*Build in processes to test and iterate*

The context-specific intervention developed in the PASSPORT study involves a set of steps that alone are simple but is it essential they work together and that appropriate support is provided throughout. It is therefore helpful to build in processes and time to test and iterate the intervention, as with any intervention development process, but with attention on keeping the intervention design focused on setting context, usability, and acceptability. The ideation workshops were a structured process for this, with the aim of developing and iterating prototypes of the tool, but this was further explored by delivering and evaluating the intervention in the pilot study. Across these processes aspects of the intervention that were initially included or developed were dropped or adapted. One example is after Workshop 1 the tool paper prototypes organised intervention activities by time frame, actioned ‘this week’, ‘this term’, and ‘this year’, to break down intervention delivery into more manageable steps and deliver some ‘quick wins’. However, during Workshop 2 and in iterating the prototype afterwards it became clear this approach did not account for the immediate actions necessary to deliver longer term activities. Ultimately, this iteration and co-design led to the action plan and milestone tracker features of the PASSPORT tool. This highlights that the successful design of context-specific interventions requires not just evidence-based or co-design elements, but an interactive testing process to ensure that it works in practice and meets the needs of all involved. This is particularly the case for complex interventions with multiple components or stages that need to work together.

## 6. Conclusion

Designing context-specific public health interventions has the potential to improve intervention success across the field. In this paper we have outlined the steps we took to design a portfolio intervention that supports schools to select and deliver tailored intervention content that would be most suitable to their setting. These steps included undertaking a range of formative studies to generate a breadth of contextual and practical insight, and then taking this insight into intervention design and content development, via co-design, school expertise, and piloting. Each of these studies or steps provided useful insight and information on the setting context and for intervention design. However, combined they provide a rich and complex grounding from which to develop a non-traditional intervention approach.

## Data Availability

**Repository: PASSPORT study formative work.** ^51^ https://doi.org/10.5523/bris.26yg5hhyaxlez24w8fhxvkrrpk This project contains the following underlying data: Scoping interviews
•
Scoping_interviews_participant_data.csv (interview participant information) • Context 1_anonymised.pdf (anonymised interview transcript) • Context 2_anonymised.pdf (anonymised interview transcript) • Context 3_anonymised.pdf (anonymised interview transcript) • Context 4_anonymised.pdf (anonymised interview transcript) • Context 5_anonymised.pdf (anonymised interview transcript) • Context 6_anonymised.pdf (anonymised interview transcript) • Context 7_anonymised.pdf (anonymised interview transcript) • Context 8_anonymised.pdf (anonymised interview transcript) • Context 9_anonymised.pdf (anonymised interview transcript) • Context 10_anonymised.pdf (anonymised interview transcript) • Context 11_anonymised.pdf (anonymised interview transcript) • Context 12_anonymised.pdf (anonymised interview transcript) • Context 13_anonymised.pdf (anonymised interview transcript) • Context 14_anonymised.pdf (anonymised interview transcript) • Context 15_anonymised.pdf (anonymised interview transcript) • Context 16_anonymised.pdf (anonymised interview transcript) •
Context_17_anonymised.pdf (anonymised interview transcript) •
Context_18_anonymised.pdf (anonymised interview transcript) • Feasibility 1_anonymised.pdf (anonymised interview transcript) •
Feasibility_2_anonymised.pdf (anonymised interview transcript) •
Feasibility_3_anonymised.pdf (anonymised interview transcript) • Feasibility_4-anonymised.pdf (anonymised interview transcript) • Feasibility_5-anonymised.pdf (anonymised interview transcript) •
Feasibility_6_anonymised.pdf (anonymised interview transcript) •
Feasibility_7_anonymised.pdf (anonymised interview transcript) •
Feasibility_8_anonymised.pdf (anonymised interview transcript) •
Feasibility_9_anonymised.pdf (anonymised interview transcript) •
Feasibility_10_anonymised.pdf (anonymised interview transcript) •
Feasibility_11_anonymised.pdf (anonymised interview transcript) •
Feasibility_12_anonymised.pdf (anonymised interview transcript) •
Feasibility_13_anonymised.pdf (anonymised interview transcript) •
Feasibility_14_anonymised.pdf (anonymised interview transcript) •
Feasibility_15_anonymised.pdf (anonymised interview transcript) • Passport information sheet_context interviews.pdf (interview participant information sheet) • Passport information sheet_feasibility interviews.pdf (interview participant information sheet) • Passport scoping interviews participant consent form.pdf (interview participant consent form template) Rapid ethnography
•
Interviews_participant_information.csv (completed observation template) •
Pupil_activities_participants.csv (completed observation template) • S1O1 Year 5 forest school.pdf (completed observation template) • S1O2 Y5 Y6 lunchtime football club.pdf (completed observation template) • S1O3 Year 5 PE external provider.pdf (completed observation template) • S1O4 Active travel.pdf (completed observation template) • S1O5 Forest School YR 5.pdf (completed observation template) • S1O6 YR 6 Girls Football Club.pdf (completed observation template) • S1O7 YR 5 PE External Provider.pdf (completed observation template) • S1O8 Lunchtime.pdf (completed observation template) • S1O9 Y6 PE with PE teacher.pdf (completed observation template) • S1O10 Active travel.pdf (completed observation template) • S1O11 mixed football after school club with PE teacher.pdf (completed observation template) • S1O12 Lunchtime Observations.pdf (completed observation template) • S1O13 After School Active Travel.pdf (completed observation template) • S1O14 YR 6 PE lesson with PE teacher.pdf (completed observation template) • S1O15 YR5 class A PE lesson with class teacher.pdf (completed observation template) • S1O16 YR5 class B PE with class teacher.pdf (completed observation template) • S1O17 Y5 PE with class teacher 1.pdf (completed observation template) • S1O18 Y5 PE with class teacher 2.pdf (completed observation template) • S1O19 Mixed football after school club with PE teacher.pdf (completed observation template) • S1O20 YR 5 class A Daily Mile.pdf (completed observation template) • S1O21 morning active travel.pdf (completed observation template) • S1O22 Y5 class 1 morning lessons.pdf (completed observation template) • S1O23 morning break.pdf (completed observation template) • S1O24 Y5 class 2 morning lessons.pdf (completed observation template) • S1O25 YR 5 & 6 after school netball club.pdf (completed observation template) • S1O26 Lunchtime girls football run by external provider.pdf (completed observation template) • S1O27 YR 5 PE Lesson with External Provider - 1.pdf (completed observation template) • S1O28 YR 5 PE Lesson with External Provider - 2.pdf (completed observation template) • S1O29 After School Dance Club Led by External Provider.pdf (completed observation template) • S1O30 morning breaktime.pdf (completed observation template) • S1O31 Y5 PE lesson with class teacher.pdf (completed observation template) • S1O32 teaching staff meeting.pdf (completed observation template) • S1O33 breakfast club morning wrap around care.pdf (completed observation template) • S1O34 Y6 class C PE with PE teacher.pdf (completed observation template) • S1O35 Y6 class B PE with PE teacher.pdf (completed observation template) • S1O36 after school wrap around childcare.pdf (completed observation template) • S1O37 YR 5 PE.pdf (completed observation template) • S1O38 YR 5 normal lesson post PE.pdf (completed observation template) • S1O39 Wrap Around Care (PM).pdf (completed observation template) • S1O40 Y5 catch up swimming lesson.pdf (completed observation template) • S1O41 Green Team Meeting.pdf (completed observation template) • S1O42 Mode Shift active travel award system.pdf (completed observation template) • S1O43 Governors meeting.pdf (completed observation template) • S1O44 Y3, Y4 and Y5 Sports Day.pdf (completed observation template) • S1O45_LSA staff meeting.pdf (completed observation template) • S1O46_bikeability.pdf (completed observation template) • S1O47 lunchtime.pdf (completed observation template) • S2O1 lunchtime observation.pdf (completed observation template) • S2O2 year 5 class observation.pdf (completed observation template) • S2O3 PE session observation.pdf (completed observation template) • S2O4 End of day travel observation.pdf (completed observation template) • S2O5 Lunchtime Observation.pdf (completed observation template) • S2O6 Year 5 Class Observation.pdf (completed observation template) • S2O7 Year 5 PE Observation.pdf (completed observation template) • S2O8 Active Travel.pdf (completed observation template) • S2O9 lunchtime observation.pdf (completed observation template) • S2O10 Year 5 PE.pdf (completed observation template) • S2O11 Active clubs.pdf (completed observation template) • S2O12 lunchtime observation.pdf (completed observation template) • S2O13 Active clubs.pdf (completed observation template) • S2O14 year 5 class observation.pdf (completed observation template) • S2O15 Year 5 PE.pdf (completed observation template) • S2O16 staff meeting.pdf (completed observation template) • S2O17 Judo club.pdf (completed observation template) • S2O18 AM AT.pdf (completed observation template) • S2O19 AM break.pdf (completed observation template) • S2O20 external club.pdf (completed observation template) • S2O21 staff meeting.pdf (completed observation template) • S2O22 Sensory circuit.pdf (completed observation template) • S2O23 School leaders meeting.pdf (completed observation template) • S2O24 enrichment led by external provider Y4.pdf (completed observation template) • S2O25 enrichment led by external provider Y2.pdf (completed observation template) • S3O1 PTA Meeting.pdf (completed observation template) • S3O2 lunchtime.pdf (completed observation template) • S3O3 afterschool travel.pdf (completed observation template) • S3O4 class pre-lunchtime.pdf (completed observation template) • S3O5 lunchtime.pdf (completed observation template) • S3O6 Y5 class after lunchtime.pdf (completed observation template) • S3O7 Y5 class pre-lunchtime.pdf (completed observation template) • S3O8 lunchtime.pdf (completed observation template) • S3O9 Y5 class after lunchtime.pdf (completed observation template) • S3O10 Y6 class pre-lunchtime inc movement break.pdf (completed observation template) • S3O11 Y6 class after lunchtime.pdf (completed observation template) • S3O12 Staff meeting.pdf (completed observation template) • S3O13 Y5 PE with PE teacher_Class B.pdf (completed observation template) • S3O14 Y5 PE with PE teacher_Class A.pdf (completed observation template) • S3O15 Before school travel.pdf (completed observation template) • S3O16 Trust Sports Day.pdf (completed observation template) • S3O17 Afternoon breaktime.pdf (completed observation template) • S3O18 Indoor PE with Y5 class 1.pdf (completed observation template) • S3O19 Indoor PE with Y5 class 2.pdf (completed observation template) • S3O20 afternoon break.pdf (completed observation template) • School 1_Activity 1_anonymised transcript.pdf (pupil activity group discussion transcript) • School 1_Activity 2_anonymised transcript.pdf (pupil activity group discussion transcript) • School 2_Activity 1_transcript.pdf (pupil activity group discussion transcript) • School 3_Activity 1_Anonymised transcript.pdf (pupil activity group discussion transcript) • S1I1_PE teacher_Transcript.pdf (anonymised interview transcript) • S1I2_chair of governors_Transcript.pdf (anonymised interview transcript) •
S1I3_external_coach_Transcript.pdf (anonymised interview transcript) •
S1I4_head_teacher_Transcript.pdf (anonymised interview transcript) •
S1I5_previous_PE_lead_Transcript.pdf (anonymised interview transcript) • S1I6_business manager_Transcript.pdf (anonymised interview transcript) •
S1I7_healthy_schools_lead_Transcript.pdf (anonymised interview transcript) •
S1I8_LSA_Transcript.pdf (anonymised interview transcript) •
S1I9_Y5_teacher1_Transcript.pdf (anonymised interview transcript) •
S1I10_PTA_Transcript.pdf (anonymised interview transcript) •
S2I1_SENCo_Transcript.pdf (anonymised interview transcript) • S2I2_External Provider_Transcript.pdf (anonymised interview transcript) • S2I3_KS2 Teacher_Transcript.pdf (anonymised interview transcript) • S2I4_PE Lead_Transcript.pdf (anonymised interview transcript) • S2I5_School Govenor_Transcript.pdf (anonymised interview transcript) • S2I6_Deputy Head_Transcript.pdf (anonymised interview transcript) • S2I7_Relevant Programme Leads_Transcript.pdf (anonymised interview transcript) •
S2I8_LSA_Transcript.pdf (anonymised interview transcript) •
S3I1_PTA_Transcript.pdf (anonymised interview transcript) • S3I2_KS2 Teacher_Transcript.pdf (anonymised interview transcript) •
S3I3_Staff_Transcript.pdf (anonymised interview transcript) • S3I4_Head Teacher_Transcript.pdf (anonymised interview transcript) • S3I5_KS2 Teacher_Transcript.pdf (anonymised interview transcript) •
S3I6_LSA_Transcript.pdf (anonymised interview transcript) • S3I7_PE Teacher_Transcript.pdf (anonymised interview transcript) • S3I8_Forest School Lead_Transcript.pdf (anonymised interview transcript) • Pupil activity assent form.pdf (pupil activity assent form template) • Pupil activity parent consent form.pdf (parent content form for pupil activity template) • Pupil activity parent information sheet.pdf (parent/carer information sheet for pupil activity) • Pupil activity pupil information sheet.pdf (pupil activity pupil information sheet) • School information sheet.pdf (school information sheet) • School study agreement form.pdf (school study agreement template) • Staff interview consent form.pdf (staff interview consent form template) • Staff interview participant information sheet.pdf (staff interview information sheet) School physical activity survey
• Passport information sheet_school survey.pdf (survey participant information sheet) • Passport school survey participant consent form.pdf (survey participant consent form template) • Codebook.csv (codebook for survey data) • Coded dataset.csv (survey dataset) •
School_demographic_information.csv (survey participant information) Co-design workshops
• Co-design workshop participant roles.csv (workshop participant information) • Co-design workshop consent form.pdf (workshop participant consent form template) • PASSPORT study co-design workshop participant information sheet.pdf (workshop participant information sheet) • CA_group design activity_anonymised transcript.pdf (workshop activity anonymised transcript) • CA_group feedback_anonymised transcript.pdf (workshop activity anonymised transcript) • CA_sketching ideas activity_anonymised transcript.pdf (workshop activity anonymised transcript) • Intervention_group design_anonymised transcript.pdf (workshop activity anonymised transcript) • Intervention_group feedback_anonymised transcript.pdf (workshop activity anonymised transcript) •
OS_discussion_anonymised transcript.pdf (workshop activity anonymised transcript) • Personas discussion_anonymised transcript.pdf (workshop activity anonymised transcript) • Tool use design_group work_anonymised transcript.pdf (workshop activity anonymised transcript) •
Tool_use_group_feedback_1_anonymised transcript.pdf (workshop activity anonymised transcript) •
Tool_use_group_feedback_2_ anonymised transcript.pdf (workshop activity anonymised transcript) •
Tool_use_group_feedback_3_anonymised transcript.pdf (workshop activity anonymised transcript) • Understanding the problem_transcript.pdf (workshop activity anonymised transcript) •
CA_transcripts_group_discussion.pdf (workshop activity anonymised transcript) •
CA_transcripts_Group_discussion_section_2 and 4.pdf (workshop activity anonymised transcript) •
CA_transcripts_Group_discussion_section_3 and 4.pdf (workshop activity anonymised transcript) •
CA_transcript_feedback.pdf (workshop activity anonymised transcript) •
CP_transcripts_exercise_group_feedback.pdf (workshop activity anonymised transcript) •
School_Porfolio_group_feedback_Transcript_anonymised.pdf (workshop activity anonymised transcript) •
School_Portfolio_group_discussion_1_Transcript_anonymised.pdf (workshop activity anonymised transcript) •
School_Portfolio_group_discussion_2_Transcript_anonymised.pdf (workshop activity anonymised transcript) •
School_Portfolio_group_discussion_3_Transcript_anonymised.pdf (workshop activity anonymised transcript) • PASSPORT Study_ Co-design workshop 3_Anonymised transcript.pdf (workshop activity anonymised transcript) There is no extended data for this study. The data can be accessed at https://doi.org/10.5523/bris.26yg5hhyaxlez24w8fhxvkrrpk. The metadata record published openly by the repository at this location clearly states how data can be accessed by bona fide researchers. Requests for access will be considered by the University of Bristol Research Data Service, who will assess the motives of potential data re-users before deciding to grant access to the data. No authentic request for access will be refused and re-users will not be charged for any part of this process. Due to the sensitivity of the data involved, whereby it was possible for school staff or pupils to reveal sensitive data about the schools or their experiences, these data are published as a restricted dataset at the University of Bristol Research Data Repository data.bris. For this reason the dataset does not carry a re-use licence in the sense of a CC-BY licence. Instead, its use is governed by the terms of the Data Access Agreement for Restricted datasets. This approach was advised by the University Research Ethics Committee as suitable in school-based studies. For more information or to arrange an MS Teams meeting please email the Research Data Service at data-bris@bristol.ac.uk. Applications must fulfil the following criteria:
• The dataset you have requested is available for sharing • You have provided verifiable institutional affiliation and contact details • You have nominated an appropriate institutional signatory (someone with the legal authority to negotiate and sign contracts on behalf of your institution) • Evidence of ethical approval (if required for the requested dataset) • Evidence of funding, commission, or sponsorship (if applicable to your study) • A summary of how you will use the requested dataset in your study • PDFs of your institutions’ data protection, data security and/or information security policies Please note the form may take between 20 to 50 minutes to complete. We will respond within 20 working days. If your application meets the criteria, a Data Access Agreement (example - http://bit.ly/2LV1OcR) is sent to your institutional signatory. Once the agreement is signed and returned, the Research Data Service team will arrange secure access to the dataset. See our webpages for accessing data via data.bris for more information: https://www.bristol.ac.uk/staff/researchers/data/accessing-research-data/ **Repository: PASSPORT study pilot.** ^52^ https://doi.org/10.5523/bris.jpczkm6e8sa82fcv2f87j5xu4 This project contains the following underlying data:
• P1_I1.pdf (anonymised interview transcript) • P1_I2.pdf (anonymised interview transcript) • P2_I1.pdf (anonymised interview transcript) • P2_I2.pdf (anonymised interview transcript) •
Pilot_interviews_IDs.csv (interview participant information) • Pupil_data.csv (pupil physical activity dataset – accelerometer, questionnaire, and demographic) • Parent carer consent form.pdf (parent/carer consent form template) • Parent carer information sheet.pdf (parent/carer information sheet) • Pupil information sheet.pdf (pupil information sheet) • School information sheet.pdf (school information sheet) • School study agreement form.pdf (school study agreement template) • Staff interview consent form.pdf (interview participant consent form template) • Staff interview participant information sheet.pdf (interview participant information sheet) There is no extended data for this study. The data can be accessed at https://doi.org/10.5523/bris.jpczkm6e8sa82fcv2f87j5xu4. The metadata record published openly by the repository at this location clearly states how data can be accessed by bona fide researchers. Requests for access will be considered by the University of Bristol Research Data Service, who will assess the motives of potential data re-users before deciding to grant access to the data. No authentic request for access will be refused and re-users will not be charged for any part of this process. Due to the sensitivity of the data involved, whereby it was possible for school staff or pupils to reveal sensitive data about the schools or their experiences, these data are published as a restricted dataset at the University of Bristol Research Data Repository data.bris. For this reason the dataset does not carry a re-use licence in the sense of a CC-BY licence. Instead, its use is governed by the terms of the Data Access Agreement for Restricted datasets. This approach was advised by the University Research Ethics Committee as suitable in school-based studies. For more information or to arrange an MS Teams meeting please email the Research Data Service at data-bris@bristol.ac.uk. Applications must fulfil the following criteria:
• The dataset you have requested is available for sharing • You have provided verifiable institutional affiliation and contact details • You have nominated an appropriate institutional signatory (someone with the legal authority to negotiate and sign contracts on behalf of your institution) • Evidence of ethical approval (if required for the requested dataset) • Evidence of funding, commission, or sponsorship (if applicable to your study) • A summary of how you will use the requested dataset in your study • PDFs of your institutions’ data protection, data security and/or information security policies Please note the form may take between 20 to 50 minutes to complete. We will respond within 20 working days. If your application meets the criteria, a Data Access Agreement (example - http://bit.ly/2LV1OcR) is sent to your institutional signatory. Once the agreement is signed and returned, the Research Data Service team will arrange secure access to the dataset. See our webpages for accessing data via data.bris for more information: https://www.bristol.ac.uk/staff/researchers/data/accessing-research-data/

## References

[1] Moore G Campbell M Copeland L : Adapting interventions to new contexts-the ADAPT guidance. *BMJ.* 2021;374:n1679. 10.1136/bmj.n1679 PMC8329746 34344699

[2] O'Cathain A Croot L Duncan E : Guidance on how to develop complex interventions to improve health and healthcare. *BMJ Open.* 2019;9(8):e029954. 10.1136/bmjopen-2019-029954 PMC6701588 31420394

[3] Skivington K Matthews L Simpson SA : A new framework for developing and evaluating complex interventions: update of Medical Research Council guidance. *BMJ.* 2021;374:n2061. 10.1136/bmj.n2061 PMC8482308 34593508

[4] Caldwell HAT Faulkner G Tremblay MS : Regional differences in movement behaviours of children and youth during the second wave of the COVID-19 pandemic in Canada: follow-up from a national study. *Can. J. Public Health.* 2022;113(4):535–546. 35507303 10.17269/s41997-022-00644-6 PMC9066998

[5] Hills AP Dengel DR Lubans DR : Supporting public health priorities: recommendations for physical education and physical activity promotion in schools. *Prog. Cardiovasc. Dis.* 2015;57(4):368–374. 10.1016/j.pcad.2014.09.010 25269062

[6] Jago R Baranowski T : Non-curricular approaches for increasing physical activity in youth: a review. *Prev. Med.* 2004;39(1):157–163. 10.1016/j.ypmed.2004.01.014 15207997

[7] Borde R Smith JJ Sutherland R : Methodological considerations and impact of school-based interventions on objectively measured physical activity in adolescents: a systematic review and meta-analysis. *Obes. Rev.* 2017;18(4):476–490. 28187241 10.1111/obr.12517

[8] Jones M Defever E Letsinger A : A mixed-studies systematic review and meta-analysis of school-based interventions to promote physical activity and/or reduce sedentary time in children. *J. Sport Health Sci.* 2020;9(1):3–17. 31921476 10.1016/j.jshs.2019.06.009 PMC6943767

[9] Love R Adams J Sluijs EMF van : Are school-based physical activity interventions effective and equitable? A systematic review and meta-analysis of cluster randomised controlled trials. *Lancet.* 2018;392:S53. 10.1016/S0140-6736(18)32174-3 PMC6563481 30628172

[10] Love R Adams J Sluijs EMF van : Are school-based physical activity interventions effective and equitable? A meta-analysis of cluster randomized controlled trials with accelerometer-assessed activity. *Obes. Rev.* 2019;20(6):859–870. 30628172 10.1111/obr.12823 PMC6563481

[11] Love RE Adams J Sluijs EMF van : Equity effects of children's physical activity interventions: a systematic scoping review. *Int. J. Behav. Nutr. Phys. Act.* 2017;14(1):134. 28969638 10.1186/s12966-017-0586-8 PMC5625682

[12] Russ LB Webster CA Beets MW : Systematic Review and Meta-Analysis of Multi-Component Interventions Through Schools to Increase Physical Activity. *J. Phys. Act. Health.* 2015;12(10):1436–1446. 10.1123/jpah.2014-0244 25599111

[13] Sluijs EM van McMinn AM Griffin SJ : Effectiveness of interventions to promote physical activity in children and adolescents: systematic review of controlled trials. *BMJ.* 2007;335(7622):703. 10.1136/bmj.39320.843947.be PMC2001088 17884863

[14] Owen CG Nightingale CM Rudnicka AR : Physical activity, obesity and cardiometabolic risk factors in 9- to 10-year-old UK children of white European, South Asian and black African-Caribbean origin: the Child Heart And health Study in England (CHASE). *Diabetologia.* 2010;53(8):1620–1630. 20454952 10.1007/s00125-010-1781-1 PMC2892063

[15] Schuch FB Stubbs B : The Role of Exercise in Preventing and Treating Depression. *Curr. Sports Med. Rep.* 2019;18(8):299–304. 10.1249/JSR.0000000000000620 31389872

[16] Skrede T Steene-Johannessen J Anderssen SA : The prospective association between objectively measured sedentary time, moderate-to-vigorous physical activity and cardiometabolic risk factors in youth: a systematic review and meta-analysis. *Obes. Rev.* 2019;20(1):55–74. 30270500 10.1111/obr.12758

[17] Bull FC Al-Ansari SS Biddle S : World Health Organization 2020 guidelines on physical activity and sedentary behaviour. *Br. J. Sports Med.* 2020;54(24):1451–1462. 10.1136/bjsports-2020-102955 PMC7719906 33239350

[18] Officers UCM: *UK Chief Medical Officers' Physical Acitivity Guidelines.* London: Department of Health and Social Care;2019.

[19] Aubert S Brazo-Sayavera J Gonzalez SA : Global prevalence of physical activity for children and adolescents; inconsistencies, research gaps, and recommendations: a narrative review. *Int. J. Behav. Nutr. Phys. Act.* 2021;18(1):81. 10.1186/s12966-021-01155-2 PMC8243483 34187486

[20] Chaput JP Willumsen J Bull F : 2020 WHO guidelines on physical activity and sedentary behaviour for children and adolescents aged 5-17 years: summary of the evidence. *Int. J. Behav. Nutr. Phys. Act.* 2020;17(1):141. 33239009 10.1186/s12966-020-01037-z PMC7691077

[21] Guthold R Stevens GA Riley LM : Global trends in insufficient physical activity among adolescents: a pooled analysis of 298 population-based surveys with 1.6 million participants. *Lancet Child Adolesc Health.* 2020;4(1):23–35. 10.1016/s2352-4642(19)30323-2 PMC6919336 31761562

[22] Jago R Salway R House D : Short and medium-term effects of the COVID-19 lockdowns on child and parent accelerometer-measured physical activity and sedentary time: a natural experiment. *Int. J. Behav. Nutr. Phys. Act.* 2023;20(1):42. 37101270 10.1186/s12966-023-01441-1 PMC10132917

[23] Jago R Salway R House D : Rethinking children’s physical activity interventions at school: A new context-specific approach. *Front. Public Health.* 2023;11. 10.3389/fpubh.2023.1149883 PMC10133698 37124783

[24] Herlitz L MacIntyre H Osborn T : The sustainability of public health interventions in schools: a systematic review. *Implement. Sci.* 2020;15(1):4. 31906983 10.1186/s13012-019-0961-8 PMC6945701

[25] Jago R Bailey R : Ethics and paediatric exercise science: issues and making a submission to a local ethics and research committee. *J. Sports Sci.* 2001;19(7):527–535. 11461056 10.1080/026404101750238980

[26] Organisation WH: *Whole-of-school approach to the promotion of physical activity: using research, policy and practice to understand system’s needs and structures.* Copenhagen;2025.

[27] Damschroder LJ Aron DC Keith RE : Fostering implementation of health services research findings into practice: a consolidated framework for advancing implementation science. *Implement. Sci.* 2009;4:50. 19664226 10.1186/1748-5908-4-50 PMC2736161

[28] Damschroder LJ Reardon CM Widerquist MAO : The updated Consolidated Framework for Implementation Research based on user feedback. *Implement. Sci.* 2022;17(1):75. 36309746 10.1186/s13012-022-01245-0 PMC9617234

[29] Jago R Porter A Salway R : PASSPORT project 1: Physical activity interventions in European primary schools for children aged 7 to 11 years of age: a scoping review protocol to create a framework of intervention components used in Europe 2023. Reference Source

[30] Porter A Walker R House D : Physical activity interventions in European primary schools: a scoping review to create a framework for the design of tailored interventions in European countries. *Front. Public Health.* 2024;12. 10.3389/fpubh.2024.1321167 PMC10883314 38389941

[31] Walker R House D Porter A : PASSPORT project 2: Exploring aspects of primary school context related to pupil physical activity and the feasibility and acceptability of the design of a tailored intervention using one-to-one interviews with school staff 2023. Reference Source

[32] House D Walker R Emm-Collison L : "We just have to work with what we've got": a qualitative analysis of contextual challenges in facilities and resources for pupil physical activity in English primary schools. *BMC Public Health.* 2025;25(1):726. 39984960 10.1186/s12889-025-21895-1 PMC11846469

[33] Walker R House D Porter A : The complexity of promoting physical activity in English state primary schools: an in-depth qualitative analysis of the role of social context. *BMC Public Health.* 2025;25(1):2912. 40855422 10.1186/s12889-025-23471-z PMC12376723

[34] House D Walker R Kent-Saisch S : PASSPORT project 3: Embedded research design for formative work for a physical activity intervention in English primary schools. 2024. Reference Source

[35] Walker R House D Kent-Saisch S : Designing context-specific physical activity interventions for English primary schools: key learning from a four-month rapid ethnography. *BMC Public Health.* 2025;25(1):2497. 40682006 10.1186/s12889-025-23682-4 PMC12273025

[36] House D Walker R Kent-Saisch S : The Value in Rapid, Multi-Site Team Ethnography for Understanding Context to Design Public Health Interventions: Learning From the PASSPORT Study. International Journal of Qualitative. *Methods.* 2025;24:24. 10.1177/16094069251401808

[37] Morgan-Trimmer S Wood F : Ethnographic methods for process evaluations of complex health behaviour interventions. *Trials.* 2016;17(1):232. 27142662 10.1186/s13063-016-1340-2 PMC4855482

[38] Walker R Emm-Collison L House D : Primary school physical activity culture in the UK: findings from a four-month rapid ethnography in three schools. *Front. Public Health.* 2026;14:1787666. 41929904 10.3389/fpubh.2026.1787666 PMC13038890

[39] Porter A House D Salway R : PASSPORT project 4: Online questionnaire exploring current primary school physical activity provision and promotion in Bristol and the wider area. 2024. Reference Source

[40] Ministry of Housing C, & Local Government: English indices of deprivation 2025. 2025. Reference Source

[41] Education Df: Schools, pupils and their characteristics Academic year 2023/24 2024. Reference Source

[42] Emm-Collison L Salway R Matthews J : Associations between the built environment, policies and curriculum in schools and primary school children’s physical activity. *Wellcome Open Research.* 2023;8:8. 10.12688/wellcomeopenres.18262.1

[43] Jago R Foster C Williams J : Assessing the impact of COVID-19 on the physical activity of Year 6 children and their parents: Identifying scalable actions to mitigate adverse impacts & provide rapid evidence to policy makers (ACTIVE-6). 2022.

[44] House D Salway R Porter A : PASSPORT Project 5: Co-design workshops to develop the PASSPORT intervention tool with primary school staff. 2024. Reference Source

[45] Friis Dam R Yu Siang T : What is Ideation – and How to Prepare for Ideation Sessions. 2024. Reference Source

[46] Abookire S Plover C Frasso R : Health Design Thinking: An Innovative Approach in Public Health to Defining Problems and Finding Solutions. *Front. Public Health.* 2020;8:459. 32984247 10.3389/fpubh.2020.00459 PMC7484480

[47] Leow MQH Moosa AS Salim H : Innovation workshop using design thinking framework and involving stakeholders to co-create ideas for management of asthma. *NPJ Prim Care Respir Med.* 2023;33(1):36. 37925475 10.1038/s41533-023-00357-4 PMC10625588

[48] Council D: The Double Diamond. 2026. Reference Source

[49] Penpot: Penpot. 2026. Reference Source

[50] House D Salway R Reid T : PASSPORT project 7: Physical Activity via a School Specific PORTfolio intervention pilot study. 2025. Reference Source

[51] Jago R House D Salway R : *PASSPORT study formative work.* Bristol Uo , editor.data.bris2026.

[52] Jago R House D Salway R : *PASSPORT study pilot.* Bristol Uo , editor.data.bris2026.

