# Supplementary material for: Designing a context-specific intervention in practice: learning from a school-based physical activity intervention
Source: NIHR Open Res. 2026 Oct 8;6:78. Originally published 2026 Jun 30. [Version 2] doi: 10.3310/nihropenres.14336.2 (PMC13680835; doi:10.3310/nihropenres.14336.2)
Supplement: Peer review history

# Reviewer report: Reviewer response for version 2

**DOI:** 10.3310/nihropenres.15783.r42833

**Published:** 2026-10-19

**Authors:** Cátia Paixão

**Affiliations:** 1 University of Aveiro, Aveiro, Portugal

I would like to thank the authors for addressing all my comments. I have no further questions. Congratulations!

Is the rationale for developing the new method (or application) clearly explained?

Yes

Is the description of the method technically sound?

Partly

Are the conclusions about the method and its performance adequately supported by the findings presented in the article?

Partly

If any results are presented, are all the source data underlying the results available to ensure full reproducibility?

Yes

Are sufficient details provided to allow replication of the method development and its use by others?

No

Reviewer Expertise:

Physical activity; respiratory diseases

I confirm that I have read this submission and believe that I have an appropriate level of expertise to confirm that it is of an acceptable scientific standard.

# Reviewer report: Reviewer response for version 2

**DOI:** 10.3310/nihropenres.15783.r42834

**Published:** 2026-10-14

**Authors:** Claire Madigan

**Affiliations:** 1 Loughborough University, Loughborough, UK

Thank you for making the changes and it is much clearer now.

Is the rationale for developing the new method (or application) clearly explained?

Yes

Is the description of the method technically sound?

Partly

Are the conclusions about the method and its performance adequately supported by the findings presented in the article?

Partly

If any results are presented, are all the source data underlying the results available to ensure full reproducibility?

Yes

Are sufficient details provided to allow replication of the method development and its use by others?

No

Reviewer Expertise:

My area of research is behavioural weight management interventions. I have developed interventions, conducted qualitative research and co-design workshops. I have also worked as a research assistant on the WAVEs study which was a school based physical activity/ dietary intervention. However I have not developed a context-specific intervention.

I confirm that I have read this submission and believe that I have an appropriate level of expertise to confirm that it is of an acceptable scientific standard.

# Reviewer report: Reviewer response for version 1

**DOI:** 10.3310/nihropenres.15620.r40938

**Published:** 2026-07-28

**Authors:** Cátia Paixão

**Affiliations:** 1 University of Aveiro, Aveiro, Portugal

**Overall comment**

 This is a methodological paper about the development of a context-specific intervention aiming to increase physical activity in primary school children. The intervention development process included interviews, an ethnographic study, a survey, and a pilot study, combined with co-design workshops with school staff.

 Overall, the paper is well-written and clearly structured, and some sections (particularly the description of the co-design workshops) present a good level of detail. However, I believe additional detail is needed in several areas to allow other researchers to replicate or adapt this approach, as the value of a methods paper like this one lies largely in its practical transferability.

**Introduction**

- Could the authors further clarify why they focused on children aged 7–11 years specifically? Is this age range based on developmental considerations (e.g. autonomy, ability to engage with active travel or breaktime activities), alignment with UK primary school key stages, precedent from the existing literature/scoping review, or a more pragmatic/logistical decision (e.g. school structure, ease of recruitment)? A brief justification would help readers understand whether this age range is a necessary feature of the approach or one that could be adapted for other age groups.

**Methodology**

- *Survey response rate*: The survey achieved a 19% response rate (59/311 schools). I think the authors could reflect more on this, as it would help readers who want to use this framework understand what barriers were faced in engaging schools, and whether the responding schools may differ systematically from non-responders (e.g. the sample skews towards urban, less deprived schools, as shown in Table 1 — was this anticipated, and how might it affect the generalizability of the context assessment items derived from this survey?). Some discussion of possible response bias and how this was accounted for (or not) in developing the context assessment would strengthen the methodology.
- *Description of the intervention*: The intervention itself is not entirely clear from the current description. Could the authors expand on: how many activities/options are typically included within each of the six pathways; what a "bespoke programme" concretely looks like once selected (e.g. how many activities does a school typically choose, over what timeframe); how prescriptive versus flexible the research team's recommendations actually are in practice; and what the relationship is between the "core package" (identical for all schools) and the bespoke, school-selected content. A concrete example or illustrative case (even briefly) of one school's context assessment leading to specific recommended activities and a final bespoke programme would substantially help readers understand how the tool operates in practice.
- *Figure 8*: The image quality of Figure 8 (the digital prototype screenshot) is poor, and much of the text within the screenshot is illegible. Could this be replaced with a higher-resolution image, or could the key elements be summarized in the accompanying text/caption to ensure the content is accessible even if the image quality cannot be improved?

**Results**

- It would be helpful if the authors linked findings from the related published papers more explicitly within this section (e.g. specific relevant findings from references 32, 33, 35, 36, 38 that directly informed particular design decisions), rather than relying mainly on citation. Even a sentence per referenced study summarizing the specific finding that shaped a specific aspect of the PASSPORT design would make this methods paper more self-contained and easier to follow without needing to consult each underlying paper separately.

Is the rationale for developing the new method (or application) clearly explained?

Yes

Is the description of the method technically sound?

Partly

Are the conclusions about the method and its performance adequately supported by the findings presented in the article?

Partly

If any results are presented, are all the source data underlying the results available to ensure full reproducibility?

Yes

Are sufficient details provided to allow replication of the method development and its use by others?

No

Reviewer Expertise:

Physical activity; respiratory diseases

I confirm that I have read this submission and believe that I have an appropriate level of expertise to confirm that it is of an acceptable scientific standard, however I have significant reservations, as outlined above.

# Response

**Published:** 2026-09-29

**Authors:** Danielle House

**Affiliations:** University of Bristol Medical School, Bristol, England, UK

Thank you very much for your helpful comments on our paper. We have addressed these and feel they have improved the clarity and focus of the paper. Details on how we addressed each of your comments are provided below.

**Overall comment**

*This is a methodological paper about the development of a context-specific intervention aiming to increase physical activity in primary school children. The intervention development process included interviews, an ethnographic study, a survey, and a pilot study, combined with co-design workshops with school staff.*

*Overall, the paper is well-written and clearly structured, and some sections (particularly the description of the co-design workshops) present a good level of detail. However, I believe additional detail is needed in several areas to allow other researchers to replicate or adapt this approach, as the value of a methods paper like this one lies largely in its practical transferability.*

**Response: Thank you, we have tried to address this and have outlined this below.**

**Introduction**

*Could the authors further clarify why they focused on children aged 7–11 years specifically? Is this age range based on developmental considerations (e.g. autonomy, ability to engage with active travel or breaktime activities), alignment with UK primary school key stages, precedent from the existing literature/scoping review, or a more pragmatic/logistical decision (e.g. school structure, ease of recruitment)? A brief justification would help readers understand whether this age range is a necessary feature of the approach or one that could be adapted for other age groups.*

**Response: Thank you for this comment. The PASSPORT study focuses on Year 5 children (aged 9-10 in the UK). This is because evidence shows that physical activity levels decline with age across childhood and the transition from primary to secondary school appears to be a key moment in this decline. This rationale for the focus on primary school broadly, and children at the end of primary school specifically, has been added to the ‘Introduction’ section.**

**Methodology**

*Survey response rate: The survey achieved a 19% response rate (59/311 schools). I think the authors could reflect more on this, as it would help readers who want to use this framework understand what barriers were faced in engaging schools, and whether the responding schools may differ systematically from non-responders (e.g. the sample skews towards urban, less deprived schools, as shown in Table 1 — was this anticipated, and how might it affect the generalizability of the context assessment items derived from this survey?). Some discussion of possible response bias and how this was accounted for (or not) in developing the context assessment would strengthen the methodology.*

**Response:** **Thank you for this suggestion. We have added reflection on the sample in the section ‘School survey’, namely that the survey was useful in many ways but demonstrated the value in triangulating data across several study designs.**

*Description of the intervention: The intervention itself is not entirely clear from the current description. Could the authors expand on: how many activities/options are typically included within each of the six pathways; what a "bespoke programme" concretely looks like once selected (e.g. how many activities does a school typically choose, over what timeframe); how prescriptive versus flexible the research team's recommendations actually are in practice; and what the relationship is between the "core package" (identical for all schools) and the bespoke, school-selected content. A concrete example or illustrative case (even briefly) of one school's context assessment leading to specific recommended activities and a final bespoke programme would substantially help readers understand how the tool operates in practice.*

**Thank you for this comment, we have expanded on the section ‘The PASSPORT intervention’ in several ways to address this:  **

- **We have added details on the assessment, recommendation, and selection process, including on the likely numbers of activities that would be recommended and selected during the process.**
- **We have added an illustrative example from a hypothetical school, to demonstrate how a context assessment would identify strengths and opportunities, how this would inform recommendations for that school, and how a school would select a number of these activities to create their bespoke programme.**
- **We have moved the information on the core package to the end of the section and expanded on this to provide additional detail, with examples of core package resources, to demonstrate how it provides guides and evidence to support the delivery of bespoke programmes.**
- **We have also provided more description and detail of the content, i.e. the materials and resources a school might be given, in the ‘Portfolio content development’ section.**

*Figure 8: The image quality of Figure 8 (the digital prototype screenshot) is poor, and much of the text within the screenshot is illegible. Could this be replaced with a higher-resolution image, or could the key elements be summarized in the accompanying text/caption to ensure the content is accessible even if the image quality cannot be improved?*

**Response: Thank you for this comment and apologies the original image quality was not sufficient. We have created a new version of this image with higher resolution.**

**Results**

*It would be helpful if the authors linked findings from the related published papers more explicitly within this section (e.g. specific relevant findings from references 32, 33, 35, 36, 38 that directly informed particular design decisions), rather than relying mainly on citation. Even a sentence per referenced study summarizing the specific finding that shaped a specific aspect of the PASSPORT design would make this methods paper more self-contained and easier to follow without needing to consult each underlying paper separately.*

**Response: Thank you for this suggestion. We have used this prompt to emphasise and bring this out across each study in the section ‘3. Developing the PASSPORT intervention’. Specifically, we have:**

- **Stated more clearly where full study findings can be accessed.**
- **Better aligned sections and language to Figure 3 which demonstrates which studies provided information on which aspects of the intervention.**
- **Expanded across each study section on the precise findings that emerged and how these informed the intervention development and design.**

# Reviewer report: Reviewer response for version 1

**DOI:** 10.3310/nihropenres.15620.r40935

**Published:** 2026-07-15

**Authors:** Claire Madigan

**Affiliations:** 1 Loughborough University, Loughborough, UK

This is a methodological paper aiming to share with other researchers about the development of a context specific intervention. There was a portfolio of interventions offered aimed to increase physical activity in primary school children and flexibility based on circumstances enables schools to choose which interventions to implement.  This project of work consisted of interview, ethnographic study, survey and pilot study to create the intervention. The studies are already published and therefore if they were read first, it would make this methodological paper easier to understand.

 Overall, it is very well written paper but there could be more detail to help researchers use this approach in future. I was uncertain until the end of the introduction of the purpose of this paper.

 Abstract

 In the abstract perhaps the aim could be the same as the introduction of using the passport intervention as a case study for the development of context- specific interventions.

 In the methods of the abstract could the authors identify that the pilot study was conducted within two schools.

 Could the methods also add more about the context specific part of the intervention as the really interesting part is the flexibility of the intervention and less about the formative work 

 Methodology

 It would be great to know how the information was synthesised together to create the Passport intervention. In Figure 4 there is a section on synthesizing findings and identify opportunities, however I couldn’t find that section in the paper. How did you do this?  Did any of the research studies have divergence, how did you take what you learned from all the work packages. What are the practical steps that were undertaken? This would be helpful to know for other researchers who want to use this approach. 

 Thinking about NIHR equality diversity and inclusion focus can you mention about the diversity of the patient and public involvement members.

 There is a lack of justification in the paper about the methodology for example why were only year 4 pupils targeted for recruitment and how were participants recruited. However, that might be because this has already been published elsewhere.  Perhaps this could be added as a sentence to each study e.g. the findings of this study have been published xxx. It might help the reader realize that the lack of detail it because it is elsewhere and this is an overview of the methodology?

 Results

 It was nice to see the learnings from the development of the intervention. It would also be helpful to better understand what worked well and what didn’t work so well. Was it hard to recruit, to keep involvement. How did you manage funding these series of studies. What were the limitations of this approach and how could we learn from this. Was it difficult to synthesize?

 Thank you for your consideration.

Is the rationale for developing the new method (or application) clearly explained?

Yes

Is the description of the method technically sound?

Partly

Are the conclusions about the method and its performance adequately supported by the findings presented in the article?

Partly

If any results are presented, are all the source data underlying the results available to ensure full reproducibility?

Yes

Are sufficient details provided to allow replication of the method development and its use by others?

No

Reviewer Expertise:

My area of research is behavioural weight management interventions. I have developed interventions, conducted qualitative research and co-design workshops. I have also worked as a research assistant on the WAVEs study which was a school based physical activity/ dietary intervention. However I have not developed a context-specific intervention.

I confirm that I have read this submission and believe that I have an appropriate level of expertise to confirm that it is of an acceptable scientific standard, however I have significant reservations, as outlined above.

# Response

**Published:** 2026-09-29

**Authors:** Danielle House

**Affiliations:** University of Bristol Medical School, Bristol, England, UK

Thank you very much for your helpful comments on our paper. We have addressed these and feel they have improved the clarity and focus of the paper. Details on how we addressed each of your comments are provided below.

**Overall comment**

*This is a methodological paper aiming to share with other researchers about the development of a context specific intervention. There was a portfolio of interventions offered aimed to increase physical activity in primary school children and flexibility based on circumstances enables schools to choose which interventions to implement. This project of work consisted of interview, ethnographic study, survey and pilot study to create the intervention. The studies are already published and therefore if they were read first, it would make this methodological paper easier to understand.*

*Overall, it is very well written paper but there could be more detail to help researchers use this approach in future. I was uncertain until the end of the introduction of the purpose of this paper.*

**Response: Thank you. Changes outlined below to the abstract have hopefully helped to clarify the purpose of the paper for the reader earlier on.**

**Abstract**

*In the abstract perhaps the aim could be the same as the introduction of using the passport intervention as a case study for the development of context- specific interventions.*

*In the methods of the abstract could the authors identify that the pilot study was conducted within two schools.*

*Could the methods also add more about the context specific part of the intervention as the really interesting part is the flexibility of the intervention and less about the formative work*

**Response: Thank you for these suggestions, we have incorporated these into the abstract.**

**Methodology**

*It would be great to know how the information was synthesised together to create the Passport intervention. In Figure 4 there is a section on synthesizing findings and identify opportunities, however I couldn’t find that section in the paper. How did you do this?  Did any of the research studies have divergence, how did you take what you learned from all the work packages. What are the practical steps that were undertaken? This would be helpful to know for other researchers who want to use this approach.*

**Response: Thank you for this comment. We have addressed it in several ways across the paper to add clarity on the intervention development process. Namely:**

- **We have expanded on how we synthesised the data from the formative studies in the section ‘Co-design of the PASSPORT tool’ and have linked this to Figure 4.**
- **Additionally, throughout the paper we have clarified and added detail on the findings that came from each formative study and how these were integrated into the intervention, with better linking to Figure 3.  **

*Thinking about NIHR equality diversity and inclusion focus can you mention about the diversity of the patient and public involvement members.*

**Response: Thank you for this suggestion. It has been complicated to report on details of information like this, as this paper is summarising work across 6 work packages which had individual processes for PPIE. To improve clarity about PPIE we have:**

- **Edited the section ‘Patient and public involvement’ to clarify that PPIE is described in published papers for relevant studies and add more detail on PPIE in the intervention development and pilot study.**
- **We moved information on our School Advisory Group from the ‘Portfolio content development’ section to the ‘Patient and public involvement’ section.**
- **PPIE contributors have not consented for us to share their demographic information. Where possible we have commented on the diversity of the schools public members came from.**

*There is a lack of justification in the paper about the methodology for example why were only year 4 pupils targeted for recruitment and how were participants recruited. However, that might be because this has already been published elsewhere. Perhaps this could be added as a sentence to each study e.g. the findings of this study have been published xxx. It might help the* *reader realize that the lack of detail it because it is elsewhere and this is an overview of the methodology?*

**Response: Thank you for this comment that has pushed us to improve clarity of methodologies for the various studies. As with PPIE, it has been complicated in this paper to include details on all studies referred to across the intervention development. To address your comment we have:  **

- **Explained the rationale for the focus on Primary school, and children at the end of primary school specifically, in the ‘Introduction’ section.**
- **Added further explanatory detail on the overall process of delivering each study in the first paragraph of section ‘3. Developing the PASSPORT intervention’.**
- **Where each formative study is described in section ‘3.1 Formative work’, we have clarified and added statements on accessing full details on participants, study processes, and findings. We have moved these to early in these paragraphs so the reader understands this from the outset.**

**Results**

*It was nice to see the learnings from the development of the intervention. It would also be helpful to better understand what worked well and what didn’t work so well. Was it hard to recruit, to keep involvement. How did you manage funding these series of studies. What were the limitations of this approach and how could we learn from this. Was it difficult to synthesize?*

**Response: Thank you for these suggestions. We have addressed these by:**

- **Adding additional detail on the PASSPORT study, including that “all formative studies, development work, pilot, and trial and evaluation were funded and delivered in this period”, to the ‘Introduction’.**

- **We have added additional information on how we synthesised data across the studies to inform the intervention development across the paper and in section ‘Co-design of the PASSPORT tool’, and have commented on the challenges of this – namely that time and a structured approach is required – in the key learning section ‘Ground intervention design in a range of data and insight’.**
- **In addition to the challenge of synthesising data, we have added further limitations to the key lessons sections, namely, the need to better trial and evaluate the levels of intervention flexibility and user autonomy, and the limit of how much testing and iteration is possible within project timeframes.**

Participant recruitment was on a study-by-study basis, and so these details are provided in published study papers. Some schools participated in more than one study, for example, all schools in the rapid ethnography study had a member of staff participate in the staff interviews study and this was a part of the recruitment approach (this is detailed in those published papers). In the main, different schools and staff participated in different studies and work packages, therefore maintaining involvement was not an issue.
